# Mismatching Expressions: Spatiotemporal and Kinematic Differences in Autistic and Non‐Autistic Facial Expressions

**DOI:** 10.1002/aur.70157

**Published:** 2026-01-18

**Authors:** Connor T. Keating, Sophie Sowden‐Carvalho, Holly O′Donoghue, Jennifer L. Cook

**Affiliations:** ^1^ School of Psychology University of Birmingham Birmingham West Midlands UK; ^2^ Department of Experimental Psychology University of Oxford Oxford UK

**Keywords:** alexithymia, autism, emotion, facial expression, social interaction

## Abstract

Preliminary studies suggest there are differences in the facial expressions produced by autistic and non‐autistic individuals. However, it is unclear what specifically is different, whether such differences remain after controlling for facial morphology and alexithymia, and whether production differences relate to perception differences. Therefore, we (1) comprehensively compared the spatiotemporal and kinematic properties of autistic and non‐autistic expressions after controlling these factors, and (2) examined the contribution of production‐related variables to emotion perception. We used facial motion capture to record 2448 cued and 2448 spoken expressions of anger, happiness, and sadness from autistic and matched non‐autistic adults. Subsequently, we extracted the activation and jerkiness of numerous facial landmarks across time, generating over 265 million datapoints. Participants also completed an emotion recognition task. Autistic participants relied more on the mouth, and less on the eyebrows, to signal anger than their non‐autistic peers. For happiness, autistic participants showed a less exaggerated smile that also did not “reach the eyes.” For sadness, autistic participants tended to produce a downturned expression by raising their upper lip more than their non‐autistic peers. Alexithymia predicted less differentiated angry and happy expressions. For non‐autistic individuals, those who produced more precise spoken expressions had greater emotion recognition accuracy. No production‐related factors contributed to autistic emotion recognition. This mismatch could explain why autistic people find it difficult to recognize non‐autistic expressions, and vice versa; autistic and non‐autistic faces may be essentially “speaking a different language” when conveying emotion.

## Lay Summary

This study compared the facial expressions produced by autistic and non‐autistic people. Our findings demonstrate that autistic and non‐autistic adults produce different angry, happy, and sad facial expressions, even after accounting for other interfering factors. This mismatch in facial expressions could explain why autistic people find it difficult to recognize non‐autistic expressions, and vice versa; autistic and non‐autistic faces may be essentially “speaking a different language” when it comes to conveying emotion. As such, what have previously been thought of as intrinsic emotion recognition “deficits” for autistic people may be more accurately described as difficulties resulting from cross‐neurotype interactions (i.e., interactions between autistic and non‐autistic people, as opposed to interactions between two autistic people). Further research is needed to test the impact of expressive differences on emotion recognition for autistic and non‐autistic people.

## 1 Introduction

Emotion recognition challenges in the autistic population are a topic of ongoing debate. Autism spectrum disorder (hereafter “autism”) is a neurodevelopmental condition, characterized by differences with social communication and interaction (American Psychiatric Association 2013). While not regarded a diagnostic feature, emotion recognition has been a focus of autism research for over three decades because it is thought that challenges in this area may contribute to putative social difficulties (Hobson 1986). Thus far, the majority of this literature has aimed to determine whether there are *differences* in emotion recognition between autistic and non‐autistic individuals [see Keating and Cook (2020)]. This work has yielded mixed findings [see Keating and Cook (2020)]: while some studies find differences in emotion recognition between groups, others find no differences, or emotion‐, task‐, or stimuli‐specific differences [e.g., in recognizing angry expressions (Ashwin et al. 2006; Bal et al. 2010; Brewer et al. 2016; Keating et al. 2022; Leung et al. 2019; Song and Hakoda 2018)]. Here, we focus on an under‐explored area: research shows that *recognizing* emotion from body movements is influenced by the way a person uses their own body to *express* emotion—here we extend this to the domain of facial expressions. We first ask whether autistic people move their faces in a different way when expressing emotions (compared to non‐autistic people); second, we question whether the production of one's own facial expressions relates to the recognition of others'.

A burgeoning body of research suggests that the way we move our own bodies affects the way we label others' body movements. For example, leveraging evidence that fast movements tend to indicate anger and slow movements indicate sadness (Michalak et al. 2009; Montepare et al. 1987; Pollick et al. 2001; Roether et al. 2009; Sawada et al. 2003), Edey and colleagues (Edey et al. 2017) showed that people who typically walk fast tend to perceive others' fast movements as less intensely angry compared to people who typically walk slow; presumably because, for fast walkers high speed movement looks relatively “normal.” Conversely, slow movers perceived fast movements as appearing intensely angry. That is, the authors showed that people use their own typical walking speed as a benchmark against which to judge the movements of others, underscoring a connection between the production and perception of whole‐body movements.

A breadth of evidence suggests that autistic individuals tend to move their bodies in different ways from non‐autistic individuals and that production differences might be linked to perception differences. Autistic individuals typically exhibit more jerky whole‐body (Nobile et al. 2011), upper limb (Anzulewicz et al. 2016; Cook, Blakemore, and Press 2013; Edey et al. 2016; Yang et al. 2014), and head (Torres and Denisova 2016) movements [see (Cook 2016)]. Importantly, (Cook et al. 2013) showed that within an autistic sample, greater jerkiness in body movements was associated with altered perception of biological motion. Autistic individuals who moved in a particularly jerky fashion were less likely to view smooth, minimally jerky, animations as “natural.” Thus, with respect to bodily movement, production differences have been linked to perception differences in the autistic population.

Preliminary evidence suggests that there are differences in the facial expressions produced by autistic and non‐autistic people [see Keating and Cook (2020) and Trevisan et al. (2018)]. The majority of this evidence comes from studies where non‐autistic observers, blind to diagnostic status, make ratings about the accuracy, quality, general appearance, and/or intensity of autistic and non‐autistic facial expressions. Autistic expressions are generally perceived to be less accurate (i.e., less socially congruous), lower in quality, and “atypical” in appearance [see Keating and Cook (2020) and Trevisan et al. (2018)], being rated as odd, awkward, or mechanical by (non‐autistic) observers (Faso et al. 2015; Grossman et al. 2013; Loveland et al. 1994; Macdonald et al. 1989). Studies have also obtained ‘intensity’ ratings, though findings are mixed with some reporting that autistic expressions are perceived to be more intense (Faso et al. 2015; Grossman et al. 2013; Lampi et al. 2023), and others less intense (Loveland et al. 1994; Legiša et al. 2013; Stagg et al. 2014; Yoshimura et al. 2015) than their non‐autistic peers. These studies—in which non‐autistic observers subjectively rate expressions—suggest that there is something different about the facial expressions produced by autistic and non‐autistic people. If this is indeed the case, then perception differences might be linked to differences in the production of emotional facial expressions.

A handful of studies have employed more objective measures to attempt to quantify the way in which facial expressions produced by autistic and non‐autistic people differ, however, a clear picture has not emerged. While some studies employing facial electromyography (fEMG) have reported reduced facial muscle activation in autism (Yirmiya et al. 1989), the majority of evidence contradicts that from subjective ratings, suggesting no differences in levels of facial muscle activation between groups (Rozga et al. 2013; Mathersul et al. 2013; Oberman et al. 2009). Notably, this lack of an effect could arise due to fEMG not being sensitive to differences in the activation of facial muscles: fEMG is typically limited to studying just two muscle groups—one responsible for frowning (corrugator supercili) and one responsible for smiling [zygomaticus major (Zane et al. 2019)]. However, while overall levels of facial muscle activation may not differ between groups, other research employing fEMG suggests that autistic children typically display less differentiated patterns of activation for positive and negative (Yirmiya et al. 1989) and happy, angry, and fearful (Rozga et al. 2013) facial expressions than their non‐autistic peers. This demonstrates that autistic individuals may produce more overlapping facial expressions across different emotions, even if mean levels of activation are similar.

An important consideration concerns facial morphology. In recent years, several studies have suggested that there may be subtle differences in facial morphology between autistic and non‐autistic individuals (Aldridge et al. 2011; Hosseini et al. 2022; Tripi et al. 2019; Tan et al. 2020). For example, using sophisticated three‐dimensional facial phenotyping, one study found that autistic people tend to have a broader mouth, upper face, and eye socket, combined with a flattened nasal bridge and shorter philtrum, relative to their non‐autistic peers (Aldridge et al. 2011). Thus, it could be that differences in the subjective appearance of expressions reflect differences in overall facial morphology (the shape and structure of the face) rather than differences in facial movement per se. Such differences in facial morphology may underpin subjective ratings of autistic expressions as odd or exaggerated (Faso et al. 2015; Grossman et al. 2013; Loveland et al. 1994; Macdonald et al. 1989; Lampi et al. 2023), because the appearance of different features contributes to judgments of facial expressions [e.g., intensity judgments; see (Diego‐Mas et al. 2020)]. Thus, any studies comparing autistic and non‐autistic facial expressions should aim to minimize the confounding influence of morphological differences.

A further issue is that alexithymia has not been accounted for in most previous research. Alexithymia comprises a subclinical condition, highly prevalent in the autistic population (Kinnaird et al. 2019), characterized by difficulties identifying and describing one's own emotions (Nemiah et al. 1976). Popular theories argue that autistic individuals' difficulties with emotion‐processing are caused by co‐occurring alexithymia, and are therefore not a feature of autism per se (Bird and Cook 2013). To date, most of the support for this hypothesis comes from studies focusing on emotion *recognition* (Cook, Brewer, et al. 2013; Milosavljevic et al. 2016; Ola and Gullon‐Scott 2020) [though see (Keating et al. 2022)]. However, alexithymia is linked to proprioceptive differences [i.e., differences in perceiving the position and movement of the body (Georgiou et al. 2016; Murphy et al. 2018; Pollatos and Herbert 2018)], and proprioception is essential for accurate motor control of both the body and the face (Bard et al. 1995; Cobo et al. 2017; Hasan and Stuart 1988; Sainburg et al. 1995). Thus, it is plausible that alexithymia could be linked to differences in the *production* of facial expressions. Indeed, there is preliminary support for this idea: Trevisan et al. (2016) identified that alexithymic, but not autistic traits, were associated with reduced expressivity of spontaneous facial expressions in autistic and non‐autistic children. As such, any study comparing emotion recognition and production in autistic and non‐autistic individuals should model the contribution of alexithymia to avoid erroneously attributing differences to autism.

In sum, it is possible that differences in the ability to recognize others' facial expressions of emotion are linked to differences in the production of those same expressions. However, progress in identifying such production differences between autistic and non‐autistic people has been hindered by methodological limitations: studies have often used low‐sensitivity methods, failed to account for facial morphology, and have not modeled the contributions of alexithymia. These limitations likely contribute to the mixed findings in the literature, particularly for voluntarily posed expressions, where results regarding the intensity (Faso et al. 2015; Loveland et al. 1994; Lampi et al. 2023; Oberman et al. 2009; Zane et al. 2019) and recognisability (Brewer et al. 2016; Faso et al. 2015; Loveland et al. 1994; Macdonald et al. 1989; Lampi et al. 2023) of facial expressions (among other factors) are highly inconsistent. To make progress, research that addresses these factors is needed.

When it comes to examining the relationship between production and perception, an important question is what features of one's own emotional expressions are likely to influence the perception of others' emotions? The body movement literature points a finger at relatively general aspects of movement: individuals who move in a more jerky manner show more extreme differences in labelling others' movements as natural (Cook, Blakemore, and Press 2013). By extension, one might predict that more jerky facial expressions are associated with reduced emotion recognition accuracy.

However, a parallel literature concerning other domains of emotion‐processing draws attention to more specific features. This literature reports that those with more precise and/or differentiated emotional experiences or visual emotion representations tend to be better at recognizing the emotions of other people (Keating and Cook 2023; Keating et al. 2023, 2026; Keating and Cook 2022). In this context, *precision* refers to how consistently a person experiences or represents a specific emotion across instances (i.e., the narrowness of the signal), while *differentiation* refers to how distinct that experience or representation is from other emotions (i.e., how far the signals are from one another). Indeed, this literature has its roots in signal detection theory [see McNicol (2005)], which argues that ‘signal’ distribution and “noise” distributions that are imprecise (i.e., wide) and indistinct (i.e., overlapping) provide low sensitivity to discriminate between the “signal” and “noise.” In the domain of emotion expression, one might predict that an individual whose angry expressions are imprecise (i.e., inconsistent) and indistinguishable from their sad expression (i.e., not differentiated) would struggle to identify other people's angry and sad expressions—perhaps because it is difficult to determine whether the incoming facial expressions matches their own angry or sad signals. Offering preliminary support for this idea, one study found that participants who were trained to produce emotional facial expressions—using automated feedback that rewarded the correct activation of facial action units—showed greater improvements in emotion recognition on an independent task, relative to an active control group (Deriso et al. 2012). Notably, those who improved the most in recognizing emotions were also the ones who showed the greatest gains in the differentiation of their own facial expressions over the course of training (Deriso et al. 2012). This provides evidence for a link between the ability to produce distinct emotional expressions and the ability to recognize those emotions in others. It also suggests a potential extension of signal detection theory—traditionally applied to perceptual discrimination—into the domain of expression production. However, further research is needed to determine whether both the precision *and* differentiation of one's own emotional facial expressions contribute to accurate recognition of others'.

### 1.1 Current Study

In the current study, we compared posed expressions of anger, happiness, and sadness from autistic and, age‐, sex‐ and IQ‐matched, non‐autistic adults, after controlling for facial morphology and alexithymia, across two conditions. Here, we focused specifically on voluntarily *posed* expressions—which are ubiquitous in everyday life, produced to deliberately communicate one's thoughts, intentions, and emotions to interaction partners (Parkinson 2005; Frith 2009; Jack and Schyns 2015)—to better characterize this type of expression within the autism literature, where findings have been mixed [e.g., (Brewer et al. 2016; Faso et al. 2015; Loveland et al. 1994; Macdonald et al. 1989; Lampi et al. 2023; Oberman et al. 2009; Zane et al. 2019)]. We included two conditions: (1) a cued condition, in which participants posed angry, happy, and sad facial expressions along to a series of audio cues, and (2) a spoken condition, in which participants posed the expressions while saying a standardized sentence. The inclusion of these two conditions was motivated by the fact that, in everyday life, we produce expressions *both* in isolation (without other concurrent movements, e.g., smiling politely while someone is talking to you), and while carrying out other movements like talking (e.g., smiling politely while talking to someone else). Despite the existence of these two types of expressions, thus far, much of the literature has solely focused on comparing ‘isolated’ posed expressions that are free from other kinds of movements. Therefore, it is unclear whether there are differences in the facial expressions posed by autistic and non‐autistic individuals when also carrying out other forms of movement (e.g., speech).

Here, to record the expressions of anger, happiness and sadness, we employed facial motion capture. Recordings were standardized to a common avatar face to minimize effects of any morphological differences, and indices were calculated representing (a) the extent of activation and (b) the jerkiness of movement, of numerous facial landmarks across time. We examined the contribution of both autism and alexithymia to differences in the expression of anger, happiness and sadness. Finally, we explored whether features of participants' own facial movements contributed to their ability to recognize others' dynamic emotional expressions.

### 1.2 Hypotheses

Based on findings that autistic people tend to exhibit more jerky whole‐body (Nobile et al. 2011), upper limb (Anzulewicz et al. 2016; Cook, Blakemore, and Press 2013; Edey et al. 2016; Yang et al. 2014), and head (Torres and Denisova 2016) movements [see Cook (2016)], we predicted that autistic participants would display significantly more jerky facial expressions than their non‐autistic counterparts. We made no formal predictions regarding the magnitude of activation of facial landmarks since this evidence was highly mixed (Faso et al. 2015; Grossman et al. 2013; Loveland et al. 1994; Macdonald et al. 1989; Lampi et al. 2023; Legiša et al. 2013; Stagg et al. 2014; Yoshimura et al. 2015; Yirmiya et al. 1989; Rozga et al. 2013), and potentially confounded by alexithymia (Keating and Cook 2020; Trevisan et al. 2016). Finally, in line with signal detection theory (McNicol 2005) and previous findings (Keating and Cook 2023; Keating et al. 2023, 2026; Keating and Cook 2022), we predicted that the precision and differentiation of participants' own productions would contribute to their ability to recognize others' facial expressions.

## 2 Method

This study was approved by the Science, Technology, Engineering and Mathematics (STEM) ethics committee at the University of Birmingham (ERN_16‐0281AP9D) and conducted in line with the principles of the revised Helsinki Declaration. All participants provided informed consent.

### 2.1 Participants

Twenty‐five autistic and 26 age‐, sex‐, and IQ‐matched non‐autistic participants were recruited from local autism research databases and through a university mailing list. Our sample size was determined through an a priori power calculation using GLIMMPSE (Kreidler et al. 2013). To have 90% power to detect a small difference between the autistic and non‐autistic participants in our outcome variables (Cohen's *d* = 0.25) at *p* < 0.05, 25 participants were required in each group, with 16 repetitions of each emotional expression (angry, happy, and sad) in each condition (cued and spoken) per participant.

All autistic participants had previously received a clinical diagnosis of autism spectrum disorder from an independent clinician. The autistic participants had significantly higher autism quotient (AQ) scores (Baron‐Cohen et al. 2001) than the non‐autistic participants (see Table 1.), with a mean AQ score comparable to large autistic population samples (e.g., 35.19) (Ruzich et al. 2015). Participants' ethnicities are reported in Supporting Information A.

**TABLE 1:** Means, standard deviations, and group differences of participant characteristics.

Variable	Non‐autistic (*n* = 26)		Autistic (*n* = 25)		Significance
Sex	16	Female	13	Female	0.512
	10	Male	11	Male	
	0	Prefer not to say	1	Prefer not to say	
Age	27.73 (10.69)		29.92 (9.67)		0.448
IQ	116.85 (13.06)		112.60 (19.88)		0.375
AQ	13.81 (7.62)		33.24 (9.13)		< 0.001
TAS	43.12 (13.58)		62.24 (12.11)		< 0.001

*Note:* In the central columns, means are followed by standard deviation in parentheses. Age is in years.

Abbreviations: AQ: autism quotient; IQ: intelligence quotient; TAS: Toronto Alexithymia Scale.

### 2.2 Community Involvement

In accordance with participatory research guidelines (Fletcher‐Watson et al. 2019; Keating 2021), members of the autism community provided feedback on our research, which led to several changes prior to data collection. For example, community members suggested dividing the 16 trials per emotion, per condition into two shorter blocks to help reduce fatigue. They also recommended that participants complete the trials for all emotions in one expression condition first (i.e., spoken angry, spoken happy, and spoken sad), and then move on to the other (i.e., cued angry, cued happy, and cued sad), to minimize the strain of repeatedly using the same facial muscles. In addition, they advised that the testing setup should accommodate both standing and seated recordings, to support participants with physical disabilities. They also recommended informing participants in advance that they would need to remove their glasses and tie back their hair if relevant—allowing individuals to prepare in ways that felt most comfortable, such as choosing to wear contact lenses or bringing their own hairbands. Several other suggestions were considered and helped shape a more accessible and inclusive study design. We carefully considered this feedback and incorporated the recommendations into our study design.

### 2.3 Procedures

Participants first completed online questionnaires and tasks assessing autistic traits [Autism Quotient (Baron‐Cohen et al. 2001)], alexithymia [Toronto Alexithymia Scale (Bagby et al. 1994)], and emotion recognition [PLF Emotion Recognition Task (Sowden et al. 2021)]. In‐lab, participants completed our FaceMap paradigm and then the two‐subtest version of the Weschler Abbreviated Scale for Intelligence (WASI‐II) (Wechsler 2011). All data were collected between January and November 2022.

#### 2.3.1 Autism Quotient

The level of autistic traits was assessed via the Autism Quotient (Baron‐Cohen et al. 2001). This self‐report questionnaire is scored on a range from 0 to 50, with higher scores representing higher levels of autistic characteristics.

#### 2.3.2 Toronto Alexithymia Scale

The level of alexithymic traits was measured via the 20‐item Toronto Alexithymia Scale (Bagby et al. 1994). The TAS comprises 20 items rated on a five‐point Likert scale (ranging from 1, strongly disagree, to 5, strongly agree). Total scores on the TAS can range from 20 to 100, with higher scores indicating higher levels of alexithymia.

#### 2.3.3 PLF Emotion Recognition Task

Participants' emotion recognition performance was assessed using the PLF Emotion Recognition Task [as in (Keating et al. 2022; Sowden et al. 2021)]. In this task, participants viewed silent, dynamic point‐light displays of the face (PLFs) which depicted actors saying sentences while expressing anger, happiness, or sadness. The audio was intentionally removed, in line with previous studies (Keating et al. 2022; Edey et al. 2017; Sowden et al. 2021), to ensure that participants relied solely on facial movements—not vocal cues—to interpret emotion. This was critical for isolating the visual component of emotional expression and examining its relationship with expression production. On each trial, after viewing each PLF video, participants rated how angry, happy, and sad the actor appeared on three visual analogue scales ranging from 0 (“Not at all angry/happy/sad”) to 10 (“Very angry/happy/sad”). Participants completed three practice trials followed by 108 randomly ordered experimental trials, across three blocks. Breaks were offered between blocks. Emotion recognition accuracy was calculated using a differentiation‐based measure: for each trial, the average of the two incorrect emotion ratings was subtracted from the correct emotion rating. This metric reflects not only whether the target emotion was identified, but also how clearly it was discriminated from the competing alternatives (see Supporting Information B for analyses using raw intensity ratings).

#### 2.3.4 FaceMap

Before starting the FaceMap paradigm, participants were instructed to remove any glasses and tie back any long hair to avoid obstruction to the face. Taking inspiration from previous research (Sowden et al. 2021), we employed the FaceMap paradigm to record facial movements during two conditions: a spoken condition and a cued condition.

In the spoken condition, participants were asked to say a standardized sentence (“my name is Jo and I'm a scientist”) while displaying the target emotion (anger, happiness, or sadness). This ensured that any speech‐related facial movements were held constant across individuals and emotions, enabling meaningful comparisons of emotional expression despite the presence of articulatory movement. Before the recordings began, participants were given the following instructions: “We will now ask you to imagine you are in a number of emotional states and to say a sentence whilst moving your face in a way which displays the facial expression for this emotion. Please imagine that you're experiencing the emotion as strongly as you can and then pose the emotion as clearly as possible. Try to think about what your own genuine expression looks like for the emotion and repeat it on each trial.” They were told the procedure would be as follows:

1. “We will ask you to imagine you are in that emotional state as strongly as you can. Tell the experimenter when you are ready.”
2. “A beep will then signal when you should start saying the sentence.”
3. “A long beep will mean the recording has finished and you can relax.”

To help participants understand the task, we showed short example videos of an actor saying a different sentence (“Today I ate cereal for breakfast”) in either a neutral or surprised manner—emotions not used in the experimental trials. Participants then completed two practice trials for each emotion (two angry, two happy, and two sad). Subsequently, they completed two experimental blocks, with participants posing eight angry, eight happy, and eight sad expressions per block (totalling 16 expressions for each emotion), counterbalanced across participants. For each trial, they were asked to imagine they were experiencing the target emotion as intensely as possible and to inform the experimenter when they felt ready. The experimenter then initiated the trial by saying “the recording is about to start” and activating a beep. Participants completed all eight trials for that block in sequence.

In the cued condition, participants were asked to pose facial expressions in response to a timed sequence of auditory cues. They were given the following instructions: “We will now ask you to imagine you are in a number of emotional states and to pose facial expressions in a different way. Please imagine that you're experiencing the emotion as strongly as you can and then pose the emotion as clearly as possible. Try to think about what your own genuine expression looks like for the emotion and repeat it on each trial.” They were then told the procedure would be as follows:

1. “We will ask you to imagine you are in that emotional state as strongly as you can.”
2. “When you are ready, listen for the first beep. At this beep, pose a neutral facial expression.”
3. “You will then hear a second, higher‐pitched beep. At this point, move your face in your own time from the neutral expression into the target facial expression we've asked you to pose.”
4. “Hold the expression until you hear a third, lower‐pitched beep, at which point return your face to a neutral expression.”
5. “A final long beep will signal that the recording has ended.”

This sequence followed a fixed timing structure: beeps were spaced at 3 s intervals, resulting in a total recording duration of 9 s per trial. Participants thus posed neutral in synchrony with the first beep, the target emotion on the second beep, returning to neutral at the third, with the recording ending after the fourth beep.

To help participants understand the task, we showed example videos of an actor posing surprised and disgusted expressions using this same timing sequence—emotions not used in the experimental trials. Participants then completed two practice trials for each of the three emotions (two angry, two happy, and two sad). They subsequently completed two experimental blocks, each comprising eight trials per emotion (anger, happiness, and sadness), counterbalanced across participants—totalling 16 trials for each emotion. As in the spoken condition, participants were asked to imagine experiencing the target emotion as strongly as possible and then to inform the experimenter when they felt ready. The experimenter then initiated the trial by saying “the recording is about to start” and activating a beep.

To facilitate the recordings, participants stood (or sat) 30 cm from an iPhone 12 mounted on a tripod with a ring light. Facial expressions were recorded using the Rokoko Face Capture tool. Rokoko employs *Apple ARKit* technology, which has been validated for facial motion tracking and is recommended for analyzing the facial movements of those with movement‐related conditions [e.g., autism (Taeger et al. 2021; Oh Kruzic et al. 2020)]. The ARKit technology employs a True Depth Camera that projects over 30,000 invisible dots to create infrared image representation of the face (Nhan 2022; Vilchis et al. 2023), which can be then used to extract levels of activation of 52 facial blendshapes, and the *X*, *Y*, and *Z* coordinates of specific landmarks. Before release, this technology was extensively tested across diverse ages and ethnicities, ensuring its suitability for tracking facial movements in individuals with varied face morphologies (Panzarino 2017).

#### 2.3.5 WASI‐II

The Intelligence Quotient (IQ) of participants was assessed via the two‐subtest version of the WASI‐II (Wechsler 2011). The two‐subtest form consists of vocabulary and matrix reasoning assessments. Scores on the WASI range from 70 to 160, with higher scores representing higher intelligence.

#### 2.3.6 Data Processing and Extraction

As discussed, preliminary literature suggests possible differences in facial morphology between autistic and non‐autistic individuals (Aldridge et al. 2011; Hosseini et al. 2022; Tripi et al. 2019; Tan et al. 2020), necessitating control for such differences when comparing emotional expressions. To address this, facial expression recordings were retargeted onto a common avatar face (using *Blender*) before data extraction (see https://osf.io/8a5yw/ for retargeting script).

To do so, we first extracted the activation of 52 facial action “blendshapes” across all timepoints by analyzing the infrared map (described above) with Apple's open‐source neural network algorithm. These blendshapes, akin to facial action units (e.g., *EyeSquintLeft*, *BrowInnerUp*, *MouthSmileLeft*), have activation scores ranging from zero (no activation) to one (peak activation). For the purposes of our statistical analyses, we used the activation data directly, but excluded the eight gaze‐related blendshapes (e.g., *eyeLookUpLeft*, *eyeLookDownRight*), leaving 44 blendshapes for analysis (see Supporting Information C for blendshape order). These data were extracted across all timepoints in the recordings—382 frames in the spoken condition and 540 in the cued condition—for the angry, happy, and sad expressions (96) of all participants (51).

We then applied the extracted activation values to animate a uniform 3D face model on Blender, ensuring that all facial movements were rendered on an identical facial structure (see https://osf.io/8a5yw/). Then, drawing inspiration from the OpenFace toolkit (Baltrušaitis et al. 2016), we defined 68 facial landmarks on the avatar (see Figure S1) and extracted their *X*, *Y*, and *Z* coordinates across time. This process ensured that all expressions were mapped onto the same facial geometry and scale, removing the influence of individual facial structure on the extracted motion features. By retargeting expressions to a common template, the displacement of facial landmarks—and therefore movement jerk at these facial landmarks—were made directly comparable across autistic and non‐autistic participants, reflecting true differences in expression dynamics rather than underlying morphological variation. After extracting these co‐ordinates, we calculated absolute jerk as the third order derivative of the raw co‐ordinates, for each of the facial landmarks (Fletcher‐Watson et al. 2019) across all timepoints (378 in spoken condition and 536 in cued condition) in the recordings. In our implementation, this involved sequentially computing movement (change in position), velocity (change in movement), acceleration (change in velocity), and then jerk (change in acceleration) from the co‐ordinate data over time. Each successive difference shortens the series by one frame, so jerk is defined over N minus four timepoints; accordingly, the first four frames of each trial do not yield valid jerk values. Here, jerk captures the smoothness or abruptness of movement, with higher values indicating more rapid changes in acceleration, and lower values reflecting smoother transitions.

By drawing inspiration from OpenFace (Baltrušaitis et al. 2016) while using the Rokoko Face Capture tool, we were able to: (1) enable comparisons with previous studies that used OpenFace; (2) conduct analyses using three‐dimensional movement data, rather than the standard two‐dimensional data typically analyzed by OpenFace; and (3) overcome the limitations of OpenFace that have been identified in prior research—specifically, difficulties in accurately tracking facial landmarks (i.e., estimating the coordinates of facial points) and in reliably estimating the activation of certain facial action units (e.g., quantifying the degree of muscle activation) (Fydanaki and Geradts 2018; Namba et al. 2021; Savin et al. 2021).

In sum, here we have two forms of data capturing different aspects of facial expression. The *blendshape data* quantify the *activation* of 44 specific groups of facial muscles (e.g., *BrowInnerUp*, *MouthSmileLeft*) across time, via values ranging from 0 (no activation) to 1 (maximum activation). The *landmark data*, in contrast, provide the *X*, *Y*, and Z coordinates of 68 fixed points on the face (e.g., corners of the eyes or mouth) across time. These coordinates were used to compute jerk (change in acceleration), capturing the smoothness of facial motion. Thus, blendshape data describe *what* movements occurred, while landmark data describe *how* those movements unfolded over time.

#### 2.3.7 Resampling Spoken Recordings

Spoken recordings were resampled using the resample() function in MATLAB to ensure uniform length for statistical comparisons. This approach uses interpolation to generate an evenly spaced time series that preserves the overall shape and temporal structure of the original signal while adjusting its length. Resampling is a widely used and valid method in time‐series analysis and has been successfully applied in prior studies involving kinematic data [e.g., Cook, Blakemore, and Press (2013) and Hickman et al. (2024)]. Notably, before resampling, there was no difference in the duration of spoken expressions between the autistic and non‐autistic participants, across all three emotions (*p* > 0.05). Resampling was not necessary for the cued condition, as all recordings were already equal in length.

#### 2.3.8 Score Calculations

As discussed, we theorized that mean levels of jerk or activation, and/or the precision (i.e., consistency of same emotional expression) and differentiation (i.e., differentiation across different emotional expressions) of one's own facial expressions could contribute to the ability to recognize others' expressions. As such, we calculated indices for each of these for both the cued and spoken expressions, in terms of both jerk and activation.

First, to get an index of the overall level of jerk and activation for cued and spoken expressions, we calculated the mean of (a) jerk and (b) activation across timepoints, landmarks, repetitions and emotions, for each condition.

Precision scores measure how consistently a person expresses the same emotion across repetitions. These scores were calculated in four steps, separately for each participant and for each emotion. First, for each of the 68 landmarks (for jerk) or 44 blendshapes (for activation), we calculated the mean value across all timepoints within each recording (i.e., for each of the 16 repetitions). Second, we computed the standard deviation of these means across the 16 repetitions, yielding a measure of variability in jerk or activation for each landmark or blendshape. Third, we calculated the average of these variability scores across all landmarks or blendshapes, resulting in a single overall variability score per emotion. Finally, we multiplied the variability score by −1 so that higher values indicated greater precision (i.e., lower variability across repetitions). This produced one precision score per emotion (anger, happiness, sadness), for both jerk and activation, which were then averaged across emotions to yield overall precision scores for the cued and spoken conditions. Higher precision scores indicate that a participant expressed an emotion in a more precise (or consistent) manner across repetitions.

Differentiation scores quantify the extent to which a person's facial expressions for one emotion differs from the facial expression for another emotion. These scores were calculated in three steps: (1) we averaged jerk and activation across repetitions and timepoints for each landmark/blendshape; (2) we calculated the absolute difference in jerk and activation between emotion pairs (angry‐happy, angry‐sad, and happy‐sad) at each landmark; and (3) we averaged these differences across landmarks to obtain a single differentiation score for each emotion pair. Finally, we averaged across emotion pairs to obtain an overall mean differentiation score for both cued and spoken expressions in terms of jerk and activation (e.g., cued jerk differentiation, cued activation differentiation, spoken jerk differentiation, and spoken jerk activation).

#### 2.3.9 Data Analysis

Our analyses comparing the facial expressions produced by autistic and non‐autistic individuals were conducted using MATLAB (version 2022b). Random forest and linear regression analyses assessing the contribution of emotion‐production factors to emotion recognition were conducted using R Studio (version 2021.09.2). Bayesian analyses were conducted in JASP (version 0.17.2.1). Heatmaps were generated using Blender (version 3.6.5). For all permutation test analyses comparing autistic and non‐autistic facial expressions (see description below), we employed an alpha of 0.05 to determine statistical significance. For all Bayesian analyses, we followed the classification scheme used in JASP (Lee and Wagenmakers 2014), in which BF_10_ values between one and three reflect weak evidence, between 3 and 10 reflect moderate evidence, greater than 10 reflect strong evidence, and greater than 100 reflect extreme evidence for the experimental hypothesis.

## 3 Results

To portray the contribution of autism and alexithymia to the production of angry, happy, and sad facial expressions across time, we rendered heatmaps (see https://osf.io/8a5yw/).

### 3.1 Analyses With Data From the Cued Condition

#### 3.1.1 Activation at the Peak of Cued Expressions

First, we aimed to determine whether there were group differences in activation during peak expression for anger, happiness, and sadness at specific blendshapes. Therefore, we extracted activation data at the midpoint of the expression (timepoint 270), for each blendshape, participant, and repetition, for each of the emotions respectively. Following this, for each of the 44 blendshapes, we conducted a linear mixed effects model (LMMs) of activation as a function of group (autistic, non‐autistic) and TAS score, with subject and repetition as random intercepts, for each of the emotions. We used linear mixed‐effects models because they account for both fixed and random effects in our nested, repeated‐measures data, preventing underestimated variance and inflated Type I‐error rates and providing more accurate, generalisable estimates (Baayen et al. 2008; Gueorguieva and Krystal 2004) (see Supporting Information D for full details and justification). In these linear mixed models, if we found a significant main effect of group, this would suggest that there are significant differences in activation between autistic and non‐autistic individuals at the specific blendshape, even after controlling for alexithymia.

To account for multiple comparisons, we implemented a permutation‐based approach (see Supporting Information D for full details). In short, for each permutation, we (1) randomly reassigned participants' activation data to the autistic or non‐autistic group, and (2) re‐ran the linear mixed effects models to generate a null distribution of F values for the group and alexithymia effects. The shuffled F values were then ranked, and the effects in the real data were only considered significant if they exceeded the 95th percentile of this null distribution. Here, permutation testing allowed us to control the family‐wise error rate while retaining greater statistical power than traditional corrections like Bonferroni, which are often overly stringent in contexts with numerous spatial and/or temporal units (e.g., neuroimaging) (Nichols and Holmes 2002; Groppe et al. 2011).

**FIGURE 1:**
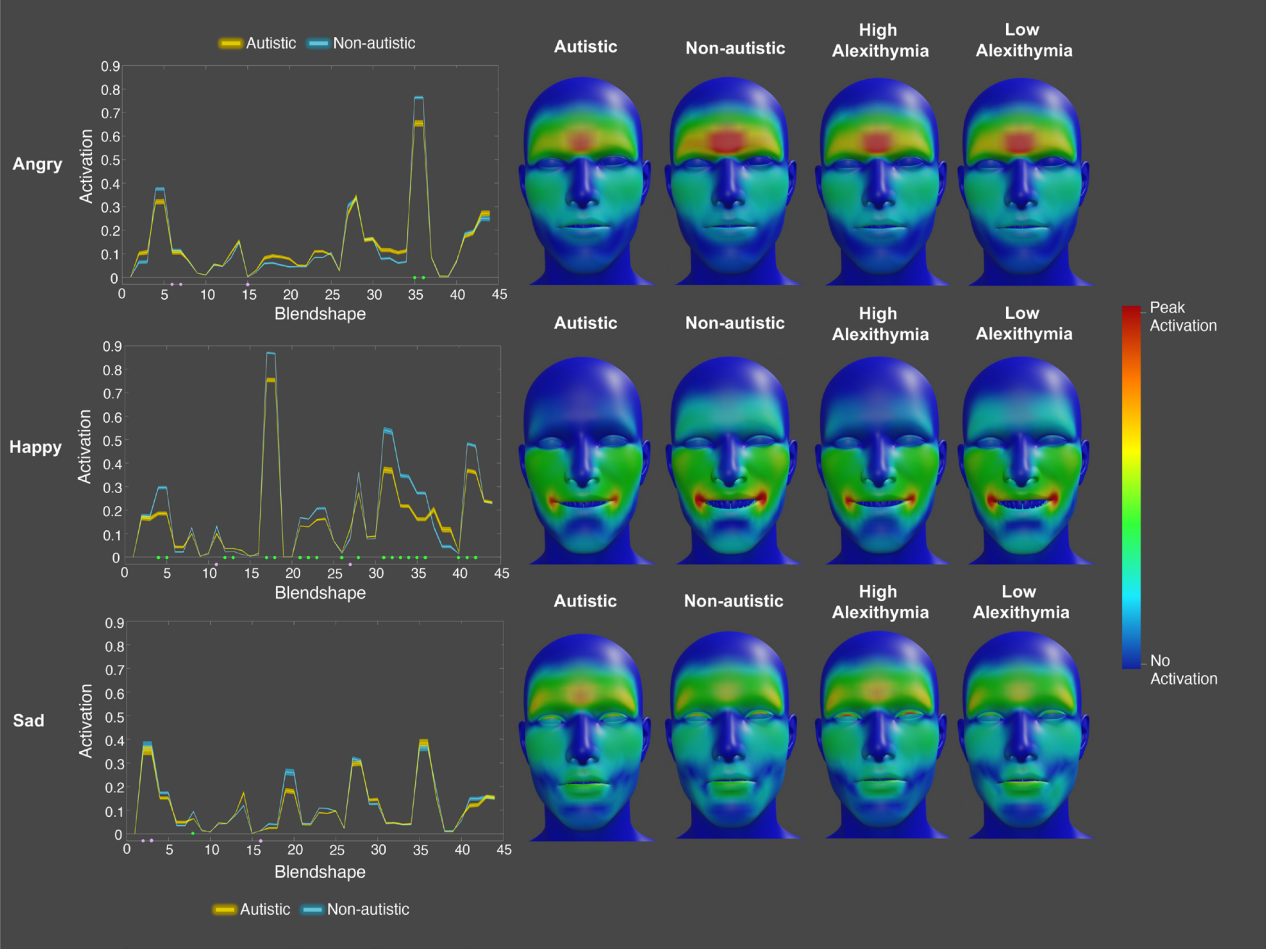
Graphs (left) and heatmaps (right) show the activation of cued angry (top), happy (middle), and sad (bottom) expressions for autistic (yellow) and non‐autistic (blue) individuals across blendshapes. Significant group effects are marked by green dots, and alexithymia effects by lilac dots. Heatmaps are standardized for each emotion.

This analysis identified that there were significant group differences in activation at specific blendshapes for the angry [4.55% of blendshapes], happy [45.55% of blendshapes] and sad [2.27% of blendshapes] expressions, even after controlling for alexithymia. When posing an angry expression, the autistic participants exhibited significantly lower activation of the left and right brow down blendshapes [left *F* = −4.91; right *F* = −4.91] – facial features typically considered to signal anger. Alexithymia was a significant negative predictor of activation for the left and right eye wide [left *F* = −5.47; right *F* = −5.49] and the left mouth [*F* = −5.47] blendshapes. For happiness, there were significant differences in activation at 45.55% of the blendshapes; the autistic participants displayed lower activation of the left and right eye squint [left *F* = −8.40; right *F* = −8.43], mouth smile [left *F* = −15.67; right *F* = −14.97], mouth dimple [left *F* = −7.84; right *F* = −6.82], mouth lower down [left *F* = −5.80; right *F* = −5.63], mouth upper up [left *F* = −8.55; right *F* = −8.31], brow down [left *F* = −7.13; right *F* = −7.12], and cheek squint [left *F* = −10.43; right *F* = −11.05] blendshapes, along with the upper mouth shrug [*F* = −5.43] and left mouth stretch [*F* = −3.72] blendshapes. Conversely, the autistic participants displayed higher activation at the upper mouth roll [F = 4.54], mouth close [F = 5.39], mouth funnel [F = 5.18], and cheek puff [*F* = 4.34] blendshapes (see Figure 1). Thus, the autistic participants displayed lower activation of many blendshapes considered to signal happiness (e.g., mouth smile, cheek squint). Alexithymia was a significant positive predictor of activation for the jaw open [*F* = 5.07] and a negative predictor of the mouth shrug lower [*F* = −5.94] blendshapes. Finally, for sadness, the autistic participants exhibited significantly lower activation for the jaw forward [*F* = −4.02] blendshape. Alexithymia was a significant positive predictor of activation for the left and right eye blink [left *F* = 5.86; right *F* = 5.94] and the right mouth [*F* = 4.02] blendshapes (see Figure 1).

#### 3.1.2 Activation Across the Time‐Course of Cued Expressions

Next, we aimed to determine whether there were any differences between groups in activation for angry, happy, and sad facial expressions at specific blendshapes and timepoints in the cued condition. To test this, for each of the 44 blendshapes, at each of the timepoints, we conducted a linear mixed effects model of activation as a function of group (autistic, non‐autistic) and TAS score, with subject and repetition as random intercepts, for each of the emotions. In these models, if we found a significant main effect of group, this would suggest that there are significant differences in activation between autistic and non‐autistic individuals at the specific blendshape, at the specific moment in time, after controlling for alexithymia. As above, we conducted a permutation test to determine which effects were statistically significant (see Supporting Information D).

**FIGURE 2:**
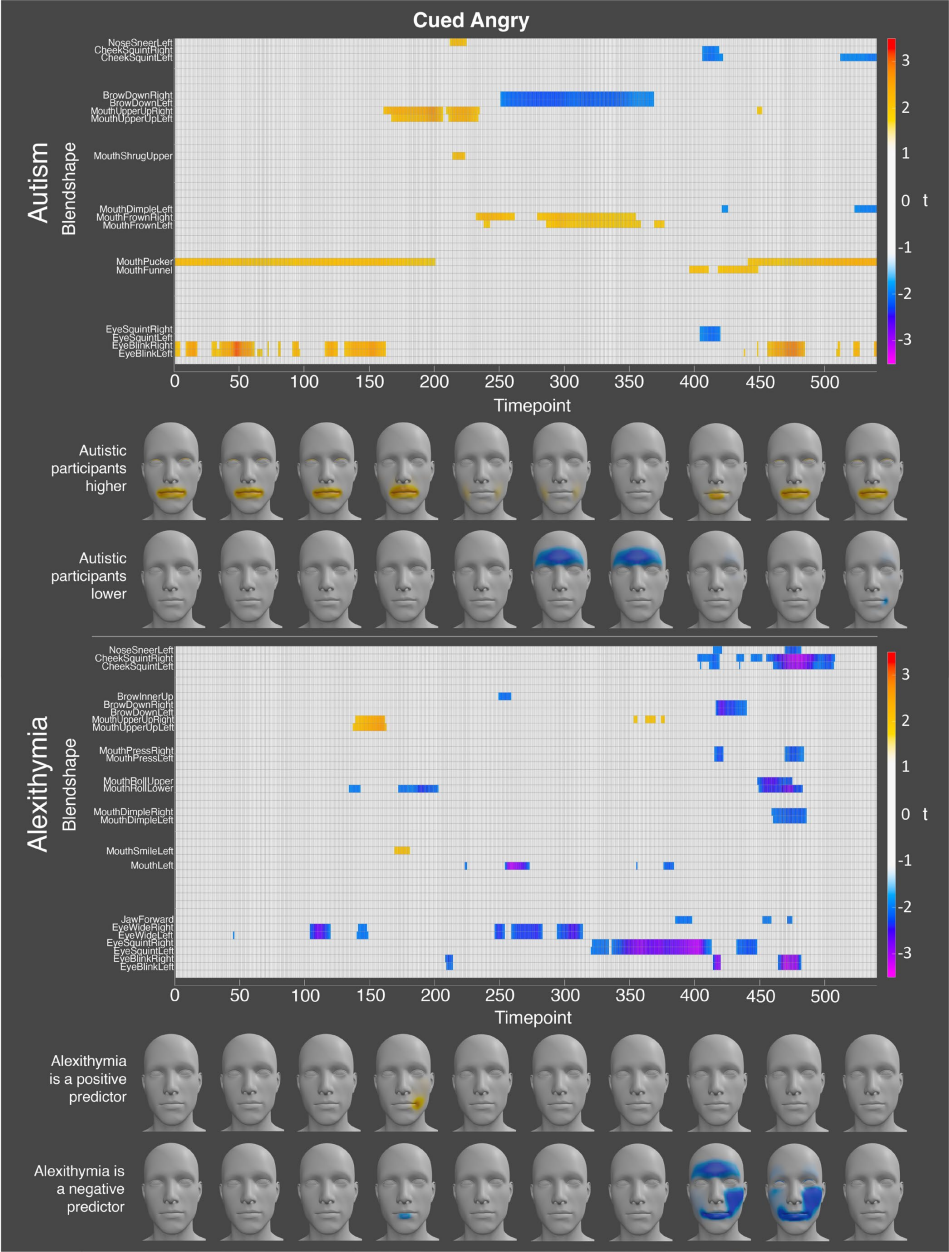
Graphs showing t‐values for significant group (top) and alexithymia (bottom) effects on activation during cued angry expressions. Positive values (orange/red) indicate higher activation in autistic participants or a positive correlation with alexithymia, while negative values (blue/purple) indicate lower activation or a negative correlation. Heatmaps are included to visualize these effects.

This analysis identified that there were significant group differences in activation for angry, happy, and sad facial expressions at specific blendshapes at specific timepoints. For anger, the autistic participants displayed significantly lower activation of the left and right brow down blendshapes for numerous timepoints when holding the angry expression (see Figure 2). In contrast, the autistic participants displayed significantly higher activation of the left and right mouth frown and mouth upper blendshapes during this period. Thus, when producing cued expressions of anger, the autistic participants may have relied more on the mouth, and less on the eyebrows, to signal anger. Prior to and after the expression, the autistic participants also displayed higher activation for the mouth pucker and left and right eye blink blendshapes. Alexithymia was a significant negative predictor of activation for the left and right eye wide and eye squint blendshapes when holding the angry expression.

**FIGURE 3:**
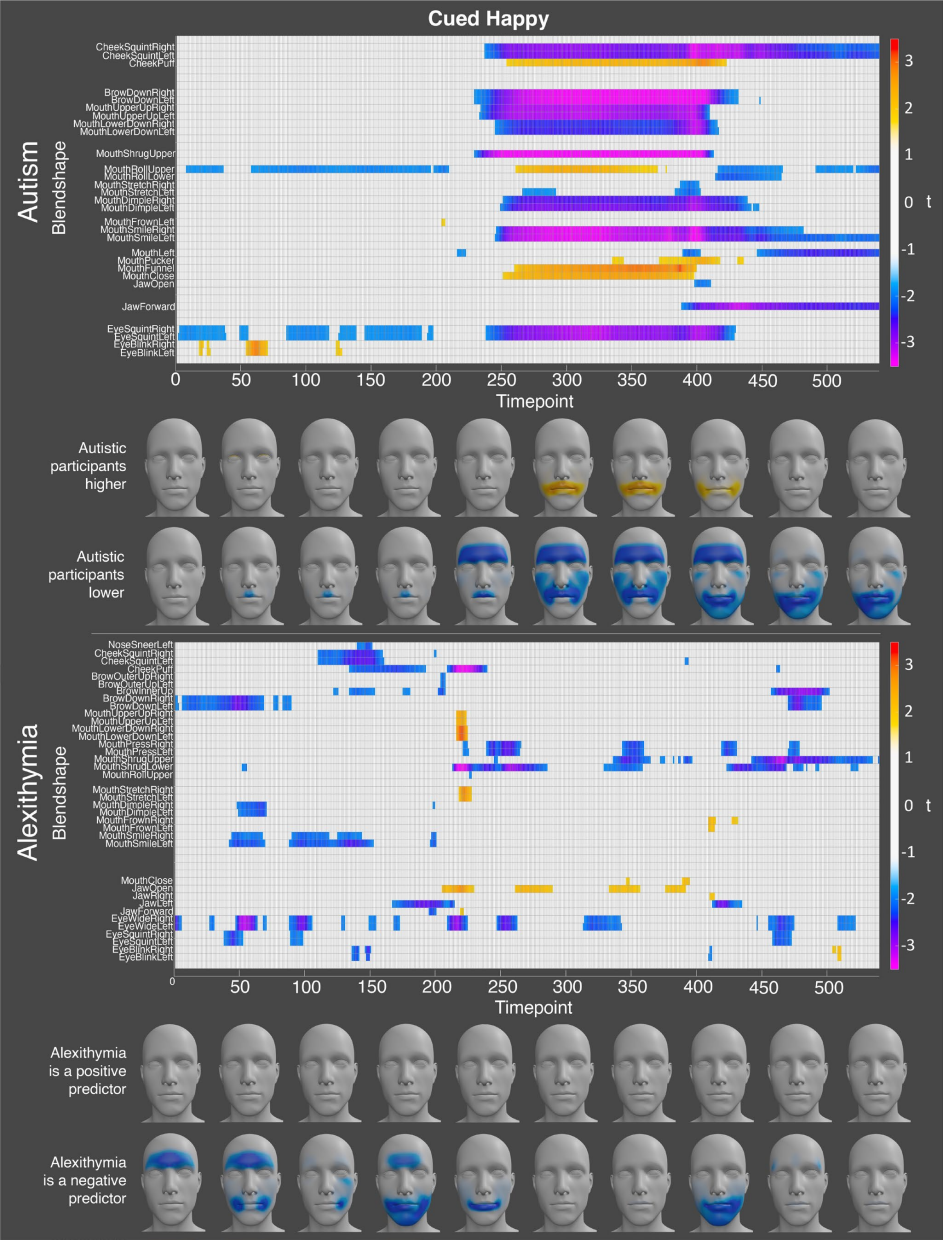
Graphs showing *t*‐values for significant group (top) and alexithymia (bottom) effects on activation during cued happy expressions. Positive values (orange/red) indicate higher activation in autistic participants or a positive correlation with alexithymia, while negative values (blue/purple) indicate lower activation or a negative correlation. Heatmaps are included to visualize these effects.

For happiness, the autistic participants displayed significantly lower activation of the left and right mouth smile, mouth dimple, mouth shrug upper, mouth lower down, mouth upper up, cheek squint, eyebrow down, and eye squint blendshapes at specific timepoints when holding the expression. In contrast, the autistic participants displayed significantly higher activation at the mouth close, mouth funnel, mouth roll upper, and cheek puff blendshapes during this period (see Figure 3). These results suggest that the autistic and non‐autistic participants display different mouth and cheek configurations when expressing happiness. Alexithymia was a significant negative predictor of activation for the left and right eye wide, mouth press, and the upper and lower mouth shrug blendshapes at peak expression. Conversely, alexithymia was a significant positive predictor of activation for the left and right mouth lower down, mouth stretch, and mouth upper up blendshapes, at timepoints immediately following the initiation of movement into the happy expression. Alexithymia was also a significant predictor of the jaw open blendshape at numerous timepoints when holding the expression (see Figure 3).

**FIGURE 4:**
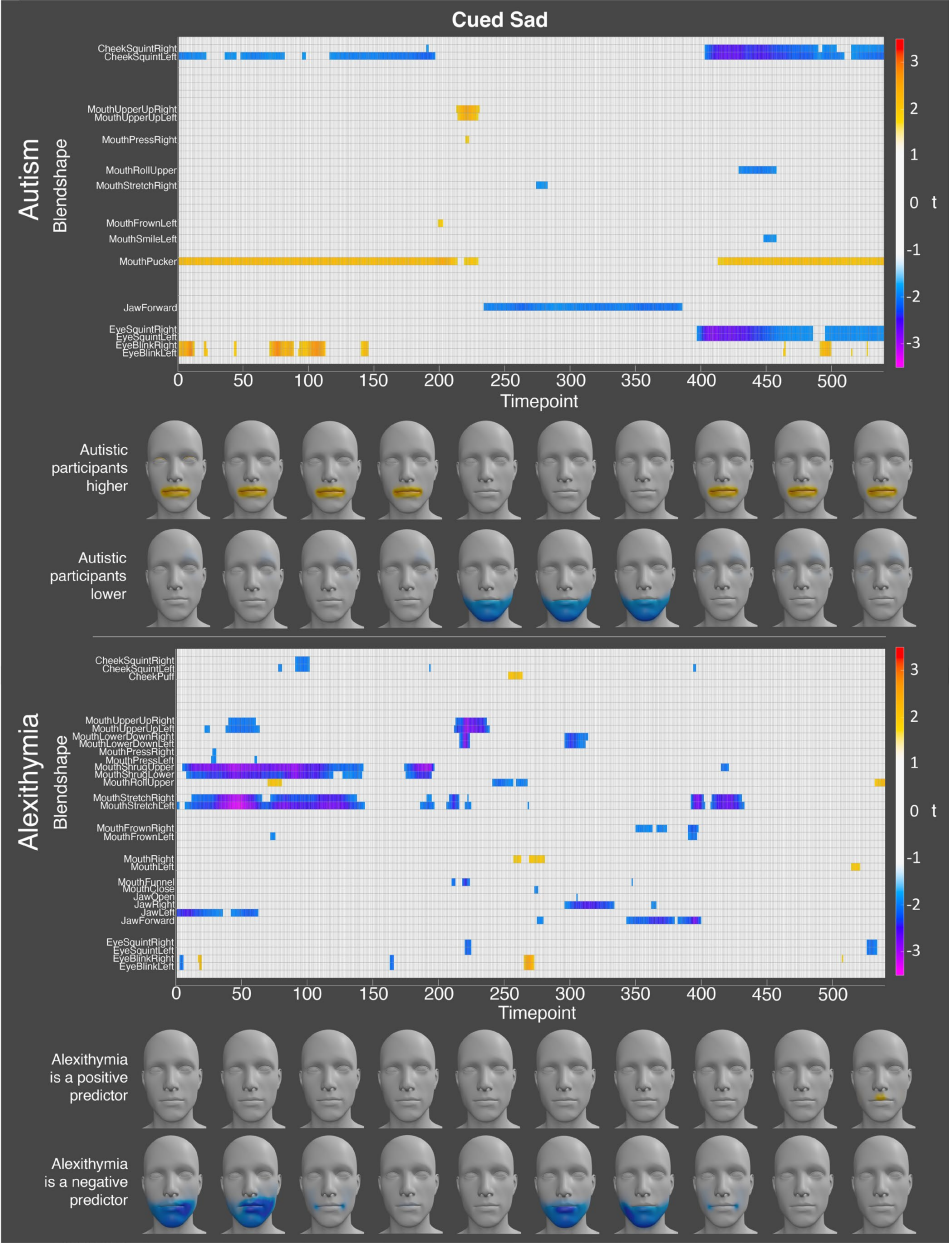
Graphs showing *t*‐values for significant group (top) and alexithymia (bottom) effects on activation during cued sad expressions. Positive values (orange/red) indicate higher activation in autistic participants or a positive correlation with alexithymia, while negative values (blue/purple) indicate lower activation or a negative correlation. Heatmaps are included to visualize these effects.

Finally, for sadness, the autistic participants displayed significantly lower activation of the jaw forward blendshape at numerous timepoints when holding the expression. In contrast, the autistic participants exhibited significantly higher activation for the left and right mouth upper up blendshapes at timepoints shortly after initiating movement into the expression (see Figure 4). Alexithymia, on the other hand, was a significant negative predictor of the left and right mouth lower down, mouth upper up, mouth stretch, the upper mouth roll, and right jaw blendshapes during this period. Conversely, alexithymia was a significant positive predictor of activation for the left and right eye blink and the mouth right blendshapes at specific timepoints when holding the expression (see Figure 4).

#### 3.1.3 Jerk Averaged Across the Whole Time‐Course of Cued Expressions

Next, we aimed to determine whether there were differences between groups in the jerkiness of cued angry, happy, and sad expressions at specific landmarks on the face. Due to previous findings that autistic individuals exhibit significantly more jerky movements, independent of movement phase [see Cook, Blakemore, and Press (2013)], we took an average of jerk across all timepoints in the recording for each landmark, participant, and repetition, for each of the emotions respectively (though see Supporting Information E for analyses comparing the jerkiness of autistic and non‐autistic facial expressions across time). Following this, for each of the 68 landmarks, we conducted a linear mixed effects model of jerk as a function of group (autistic, non‐autistic) and TAS scores, with subject and repetition as random intercepts for each of the emotions. As previously, we conducted a permutation test on the data to account for multiple testing (see Supporting Information D).

**FIGURE 5:**
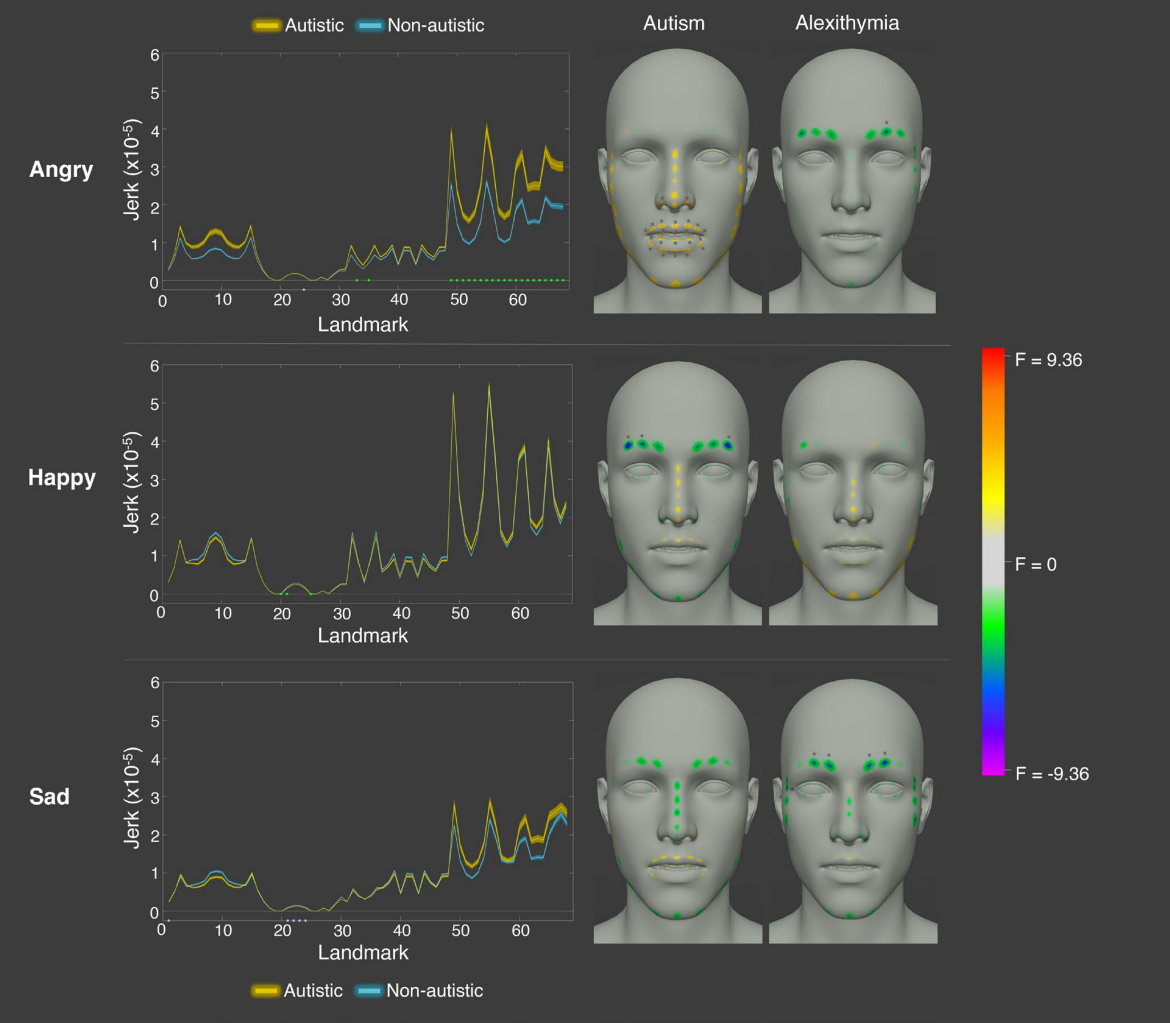
Graphs showing jerkiness of cued angry (top), happy (middle), and sad (bottom) facial movements for autistic (yellow) and non‐autistic (blue) participants across landmarks. Significant group differences are marked in green, and alexithymia effects in lilac. The right panel shows F values for group and alexithymia effects at each landmark. Positive values (yellow, orange, red) indicate higher jerk in autistic participants or a positive correlation with alexithymia; negative values (green, blue, and purple) indicate lower jerk or a negative correlation. Stars denote statistical significance (*p* < 0.05).

This analysis revealed that there were significant group differences in jerk for angry and happy (but not sad) expressions at specific regions on the face (note that the largest number of significant differences were found for anger = 32.35% landmarks; happiness = 4.41% landmarks). When posing angry expressions, the autistic participants exhibited significantly higher jerk than the non‐autistic participants at all of the mouth facial landmarks [mean significant *F* = 4.19] and at specific nose landmarks [22.2% nose landmarks; mean significant *F* = 3.71], even after controlling for alexithymia (see Figure 5). Alexithymia, on the other hand, was a significant negative predictor at specific eyebrow landmarks [10% eyebrow landmarks; mean significant *F* = −3.87]: those higher in alexithymia exhibited lower jerk at a specific eyebrow landmark when posing anger (see Figure 5). In contrast, for happiness, the autistic participants displayed significantly *lower* jerk at a third of the eyebrow landmarks (33.33% eyebrow landmarks; mean significant *F* = −7.62). It is likely that the autistic participants displayed significantly lower jerk at the eyebrow region due to there being lower activation of the left and right ‘eyebrow down’ blendshapes, as per our previous analysis. Alexithymia was not a significant predictor of jerk at any of the landmarks when posing happiness. Finally, there were no significant group differences in jerk for sad expressions at any of the facial landmarks. Nevertheless, alexithymia was a significant negative predictor of jerk at specific eyebrow [40% eyebrow landmarks; mean significant *F* = −4.56] and jaw [5.88% jaw landmarks; *F* = −3.76] landmarks: those higher in alexithymia exhibited lower jerk at specific eyebrow landmarks when posing sadness (see Figure 5).

### 3.2 Analyses With Data From the Spoken Condition

#### 3.2.1 Activation Averaged Across the Whole Time‐Course of Spoken Expressions

Next, we aimed to determine whether there were group differences in activation for the angry, happy, and sad *spoken* expressions at specific blendshapes. In this condition, since the expression was produced across the whole recording, we took an average of activation across all timepoints for each blendshape, participant, and repetition, for each of the emotions respectively. Following this, for each of the 44 blendshapes, we conducted a linear mixed effects model of activation as a function of group (autistic, non‐autistic) and TAS score, with subject and repetition as random intercepts, for each of the emotions. To account for multiple testing, we conducted a permutation test (see Supporting Information D).

**FIGURE 6:**
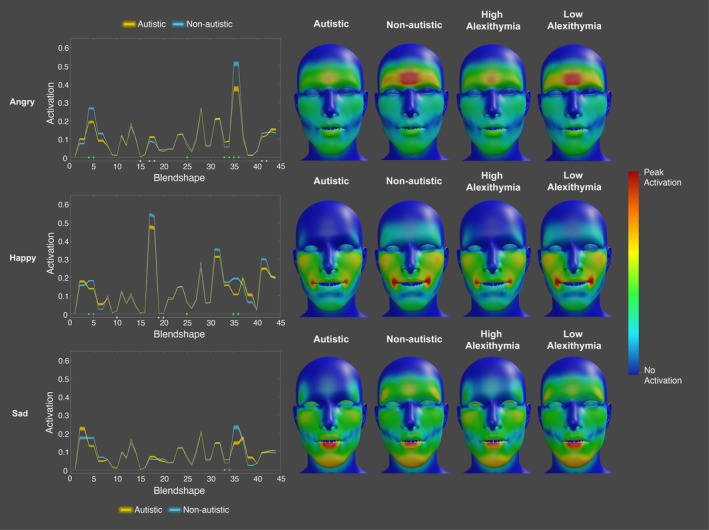
Graphs (left) and heatmaps (right) show the activation of spoken angry (top), happy (middle), and sad (bottom) expressions for autistic (yellow) and non‐autistic (blue) individuals across blendshapes. Significant group effects are marked by green dots, and alexithymia effects by lilac dots. Heatmaps are standardized for each emotion.

This revealed that there were significant group differences in activation for spoken expressions of anger [15.91% of blendshapes], happiness [11.36% of blendshapes] and sadness [4.55% blendshapes]. For anger, the autistic participants displayed significantly lower activation of the left and right eye squint [left *F* = −4.93; right *F* = −4.91], brow down [left *F* = −3.70; right *F* = −3.70], and the mouth roll upper [*F* = −5.90] blendshapes, and significantly higher activation of the left and right mouth upper up [left *F* = 3.94; right *F* = 4.70] blendshapes. Thus, across both the cued and spoken condition, the autistic participants displayed lower activation of the brow down blendshapes. Notably, alexithymia was a significant positive predictor of activation at the left and right mouth smile [left *F* = 8.30; right *F* = 8.97], cheek squint [left *F* = 4.21; right *F* = 3.82], and left mouth [*F* = 6.88] blendshapes (see Figure 6). Hence, those high in alexithymic traits showed increased activation of many of the blendshapes associated with happiness (mouth smile, cheek squint) when posing anger, suggesting that these facial expressions may be less well differentiated. For happy spoken expressions, the autistic participants exhibited significantly lower activation of the left and right eye squint [left *F* = −4.12; right *F* = −4.12], brow down [left *F* = −10.45; right *F* = −10.42], and the mouth roll lower [*F* = −3.78] blendshapes (see Figure 6). Thus, across both the cued and spoken condition, the autistic participants displayed lower activation of the brow down blendshapes when expressing happiness. In addition, alexithymia was a significant positive predictor of the left and right mouth frown blendshapes [left F = 5.35; right *F* = 5.91], and a significant negative predictor of activation for right jaw blendshape [*F* = −4.39]. Hence, when posing happiness, those high in alexithymic traits showed increased activation of some of the blendshapes associated with anger (e.g., mouth frown), suggesting once again that their happy expressions may be less well‐differentiated from their angry expressions. Finally, for sad spoken expressions, the autistic participants displayed higher activation of the left and right mouth upper up [left *F* = 8.39; right *F* = 9.63] blendshapes (see Figure 6). Alexithymia was not a significant predictor of activation for sad spoken expressions at any of the blendshapes.

#### 3.2.2 Activation Across the Time‐Course of Spoken Expressions

Next, we aimed to determine whether there were any differences between groups in activation for angry, happy, and sad facial expressions at specific blendshapes, at specific timepoints in the spoken expression, after controlling for alexithymia. To test this, for each of the 44 blendshapes, at each of the timepoints, we conducted a linear mixed effects model of activation as a function of group (autistic, non‐autistic) and TAS score, with subject and repetition as random intercepts, for each of the emotions. As above, we employed a permutation test to establish which effects were statistically significant (see Supporting Information D).

**FIGURE 7:**
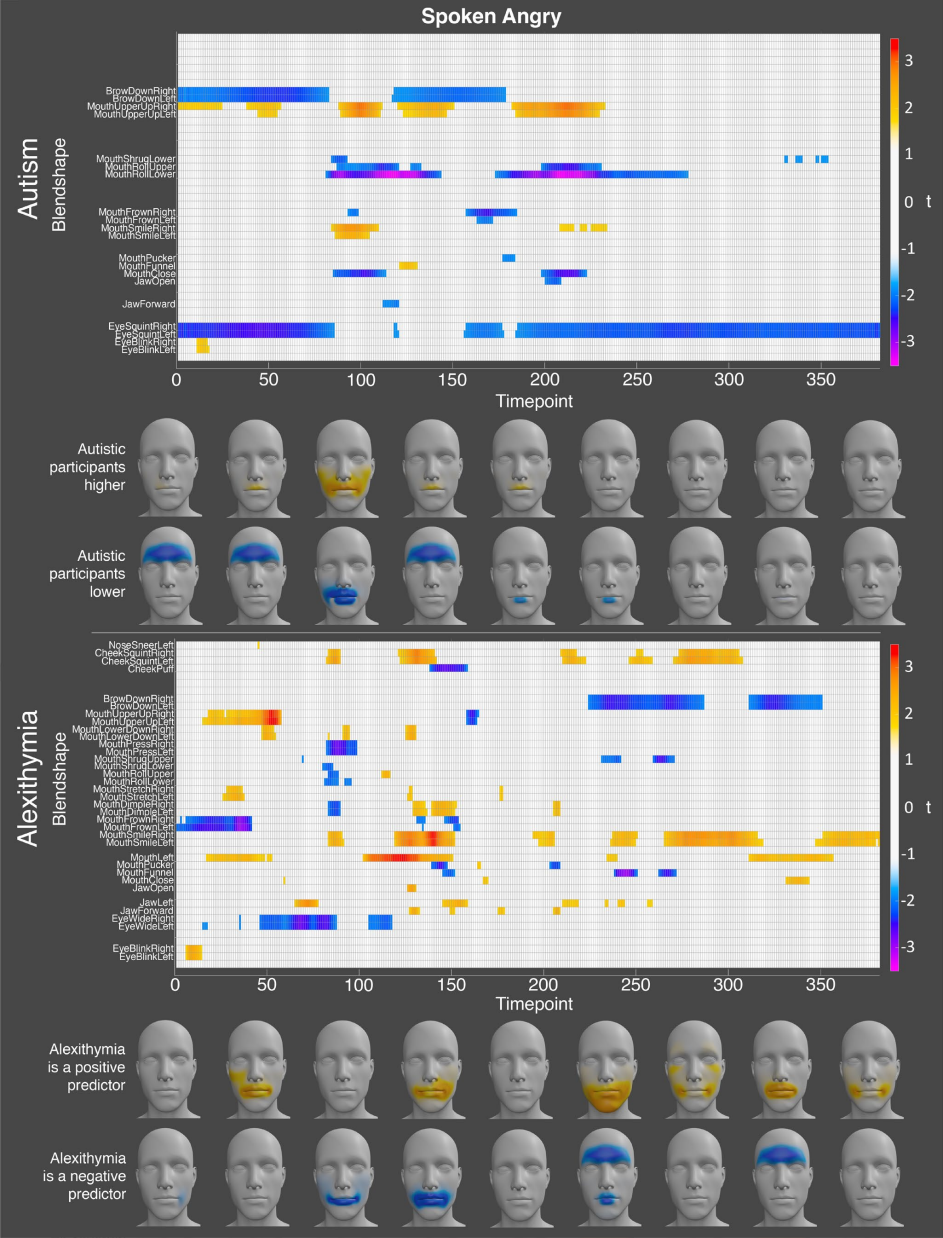
Graphs showing *t*‐values for significant group (top) and alexithymia (bottom) effects on activation during spoken angry expressions. Positive values (orange/red) indicate higher activation in autistic participants or a positive correlation with alexithymia, while negative values (blue/purple) indicate lower activation or a negative correlation. Heatmaps are included to visualize these effects.

This analysis revealed that, for anger, the autistic participants displayed significantly lower activation of the left and right brow down and eye squint, the lower and upper mouth roll, and the mouth close blendshapes at numerous timepoints throughout the expression. In contrast, the autistic participants displayed significantly higher activation of the left and right mouth upper up blendshapes at numerous timepoints throughout, and the mouth smile blendshapes early in the angry expression. Alexithymia was a significant positive predictor of the left and right mouth smile and cheek squint blendshapes at numerous timepoints throughout the angry expression, thus suggesting that angry and happy expressions are less well differentiated for highly alexithymic individuals. In comparison, alexithymia was a significant negative predictor of the brow down blendshapes later in the expression (see Figure 7 for all significant differences).

**FIGURE 8:**
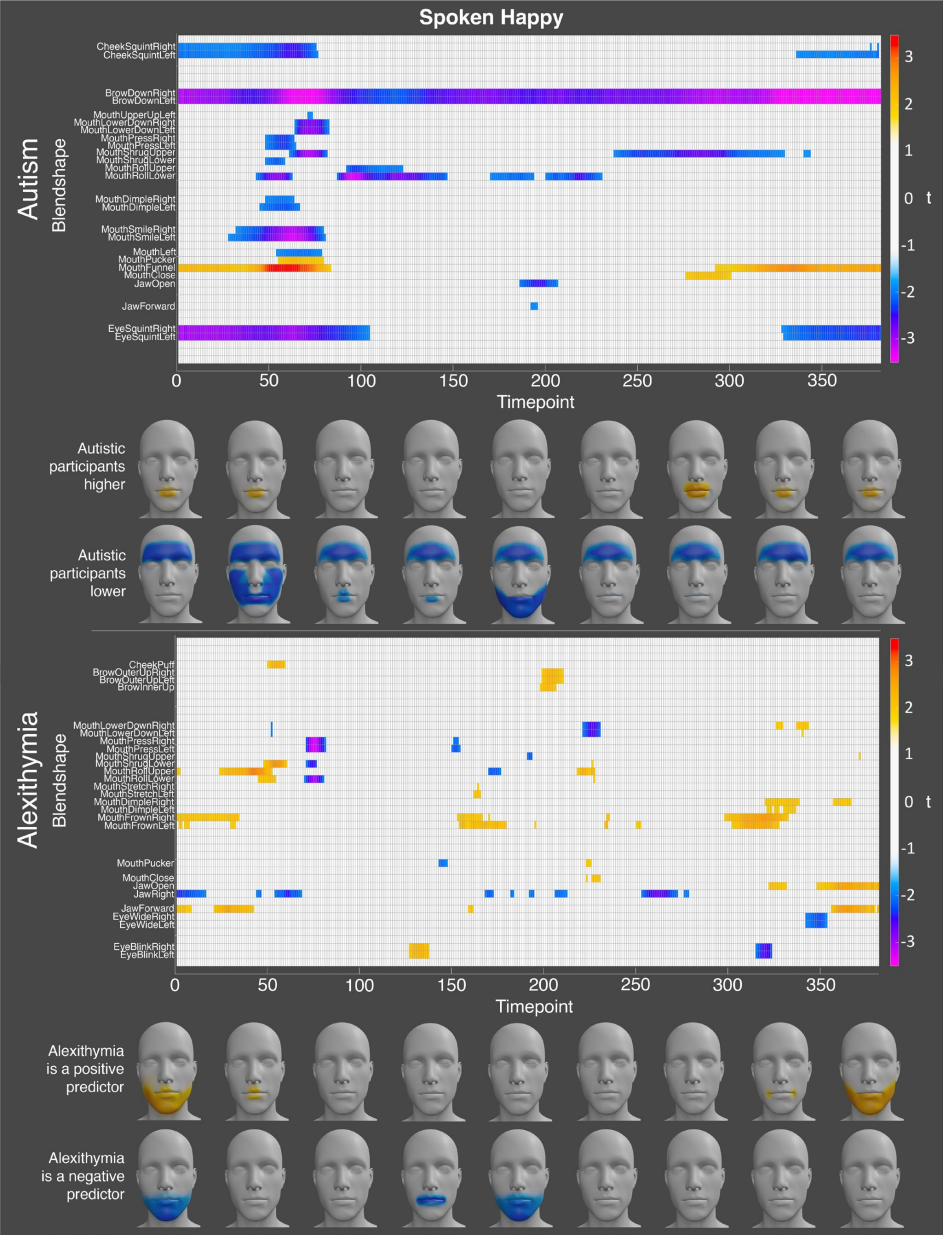
Graphs showing *t*‐values for significant group (top) and alexithymia (bottom) effects on activation during spoken happy expressions. Positive values (orange/red) indicate higher activation in autistic participants or a positive correlation with alexithymia, while negative values (blue/purple) indicate lower activation or a negative correlation. Heatmaps are included to visualize these effects.

For happiness, the autistic participants exhibited significantly lower activation of the left and right brow down blendshapes at every timepoint in the recording. In addition, the autistic participants displayed significantly lower activation of the left and right cheek squint, eye squint, and mouth shrug upper blendshapes at the start and end of the expression, and many of the mouth‐related blendshapes (e.g., left and right mouth lower down, mouth smile, mouth dimple, mouth press, etc.) at the start of the expression. In contrast, the autistic participants displayed higher activation of the mouth pucker and mouth funnel blendshapes at the start and end of the expression. Alexithymia, on the other hand was a significant positive predictor of the left and right mouth frown blendshapes at numerous timepoints throughout, suggesting that highly alexithymic individuals tend to activate action units associated with anger when expressing happiness (see Figure 8 for all significant effects).

**FIGURE 9:**
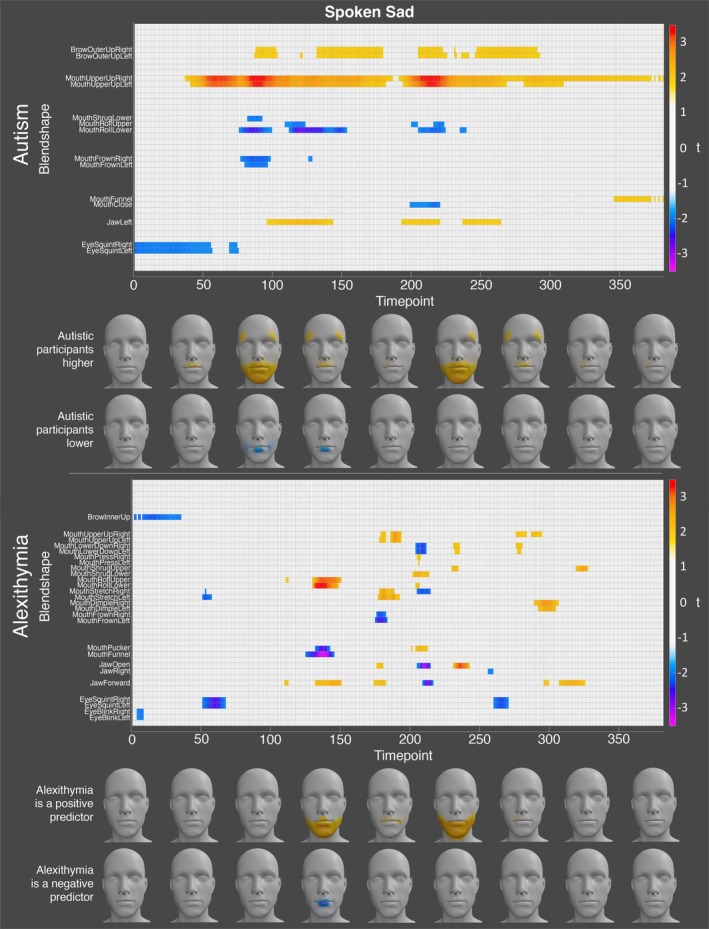
Graphs showing *t*‐values for significant group (top) and alexithymia (bottom) effects on activation during spoken sad expressions. Positive values (orange/red) indicate higher activation in autistic participants or a positive correlation with alexithymia, while negative values (blue/purple) indicate lower activation or a negative correlation. Heatmaps are included to visualize these effects.

Finally, for sadness, the autistic participants displayed significantly higher activation of the left and right mouth upper up and brow outer up blendshapes, along with the left jaw blendshape, at numerous timepoints throughout the expression. Concurrently, the autistic participants exhibited significantly lower activation of the upper and lower mouth roll, and lower mouth shrug blendshapes throughout the expression. Finally, the autistic participants displayed lower activation of the left and right eye squint and mouth frown blendshapes near the start of the expression (see Figure 9). Alexithymia was a significant positive predictor of the upper and lower mouth roll and mouth shrug blendshapes, and the left and right mouth upper up and mouth dimple blendshapes at various timepoints throughout the expression. Alexithymia was a significant negative predictor of the left and right eye squint blendshapes at the start and end of the expression, and of the mouth pucker and mouth funnel blendshapes near the start of the expression (see Figure 9).

#### 3.2.3 Jerk Averaged Across the Whole Time‐Course of Spoken Expressions

Finally, we aimed to determine whether there were significant group differences in the jerkiness of spoken expressions across the emotions. To fulfill this aim, we took an average of jerk across all timepoints in the recording for each landmark, participant, and repetition, for each of the emotions respectively (see Supporting Information E for analyses comparing the jerkiness of autistic and non‐autistic facial expressions across time). Following this, for each of the 68 landmarks, we conducted a linear mixed effects model of jerk as a function of group (autistic, non‐autistic) and TAS scores, with subject and repetition as random intercepts, for each of the emotions. As previously, we conducted a permutation test on the data to account for multiple testing (see Supporting Information D).

**FIGURE 10:**
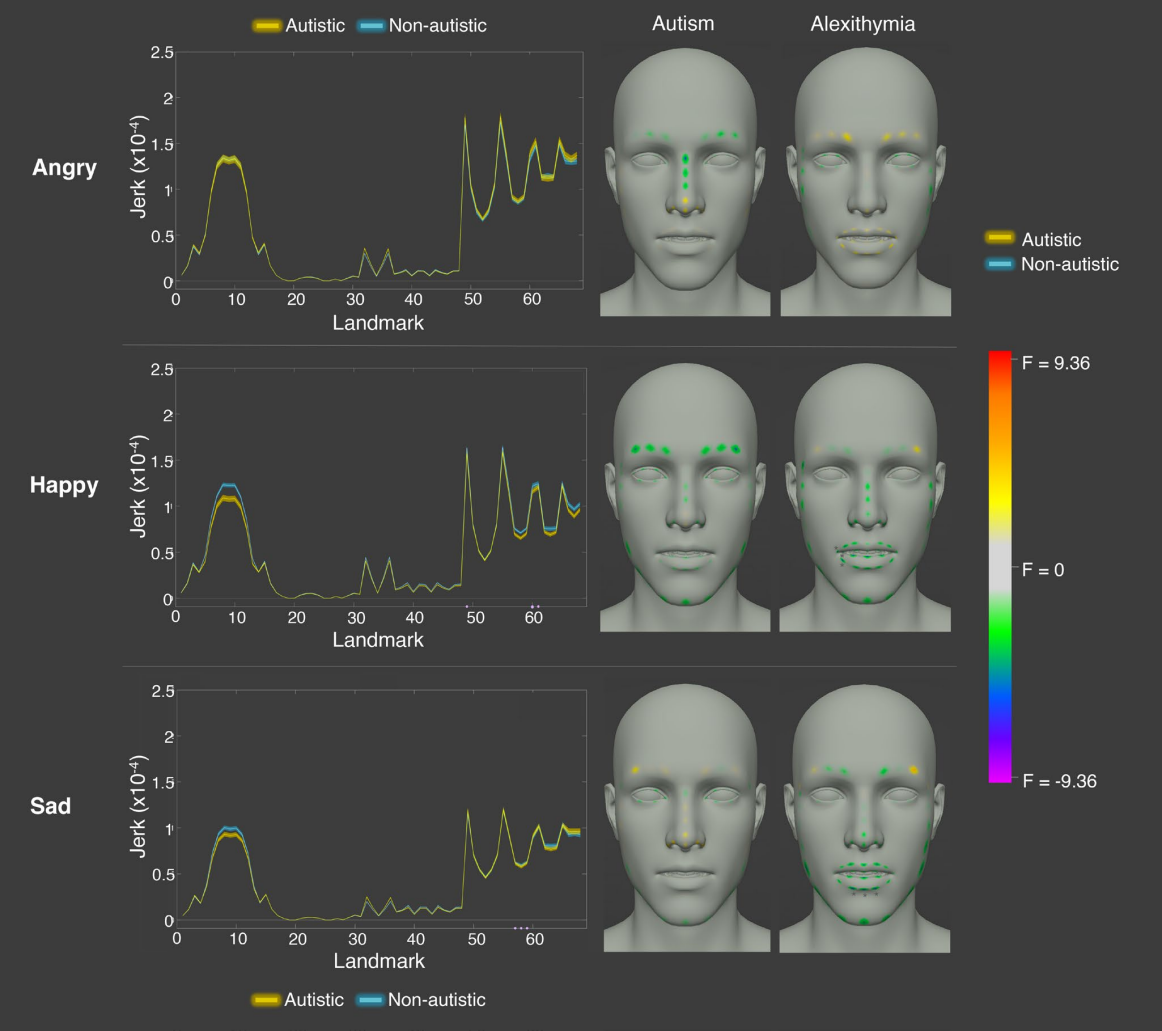
Graphs showing jerkiness of spoken angry (top), happy (middle), and sad (bottom) facial movements for autistic (yellow) and non‐autistic (blue) participants across landmarks. Significant group differences are marked in green, and alexithymia effects in lilac. The right panel shows F values for group and alexithymia effects at each landmark. Positive values (yellow, orange, red) indicate higher jerk in autistic participants or a positive correlation with alexithymia; negative values (green, blue, purple) indicate lower jerk or a negative correlation. Stars denote statistical significance (*p* < 0.05).

Our analysis revealed that there were no significant group differences in the jerkiness of movements for angry, happy, or sad spoken expressions at any of the facial landmarks. Similarly, alexithymia did not predict jerk at any of the facial landmarks for angry expressions. However, for happiness and sadness, alexithymia was a negative predictor of jerk at specific mouth facial landmarks [happiness: 15% mouth landmarks; mean significant *F* = −4.41; sadness: 15% mouth landmarks, mean significant *F* = −4.45; see Figure 10].

#### 3.2.4 The Differentiation of Angry and Happy Facial Expressions: Exploratory Analysis

As discussed previously, the results from our primary analyses raise the possibility that those high in alexithymia produce less differentiated angry and happy facial expressions than those low in alexithymia, even after accounting for autism. That is, we found that individuals high in alexithymia displayed elevated activation of the mouth smile blendshapes when posing anger, and the mouth frown blendshape when posing happiness (relative to those low in alexithymia). Thus, to formally test the contribution of autism and alexithymia to the differentiation of angry and happy spoken expressions, we conducted an exploratory random forests analysis (Breiman 2001) using the Boruta wrapper algorithm (Kursa and Rudnicki 2010).

**FIGURE 11:**
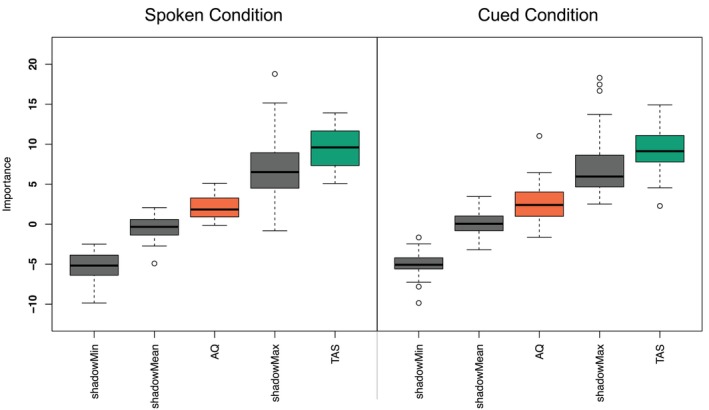
Random forest importance scores for AQ and TAS in differentiating spoken (left) and cued (right) angry and happy expressions. Boxplots display variable importance, with box edges representing the interquartile range (IQR), whiskers extending 1.5 * IQR from the edges, and circles indicating outliers. Box color indicates decision: Green (confirmed), Yellow (tentative), Red (rejected). Gray represents meta‐attributes (shadowMin, shadowMax, and shadowMean).

In this analysis, alexithymia was deemed important [Mean Importance Score (MIS) = 9.59], and autism was deemed unimportant [MIS = 2.13], for the differentiation of angry and happy *spoken* expressions (see Figure 11, left). A follow‐up analysis identified the same pattern of results in the cued condition (alexithymia [MIS = 9.36]; autism [MIS = 2.64]; see Figure 11, right). In sum, these results suggest that alexithymia, and not autism, is associated with lower differentiation of angry and happy facial expressions across both posing conditions.

#### 3.2.5 The Link Between Production and Perception

Subsequently, we aimed to investigate whether features of emotion‐production contribute to emotion recognition accuracy. Building on the body movement and emotional experience literatures, we predicted that more jerky, and less precise and/or differentiated, expressions would be associated with reduced emotion recognition accuracy. We explored whether this was the case for both autistic and non‐autistic individuals by conducting a random forests analysis (Breiman 2001) separately for each group, using the Boruta wrapper algorithm (Kursa and Rudnicki 2010) [as in (Keating and Cook 2023; Keating et al. 2023, 2026)]. Here, we analyzed the groups separately because prior research suggests that different abilities or processes may underlie emotion recognition in autistic and non‐autistic individuals (Keating et al. 2023, 2026; Rump et al. 2009; Rutherford and McIntosh 2007; Walsh et al. 2014). To test our predictions, we included emotion recognition accuracy as the outcome variable: feature variables included mean jerk, mean jerk precision, mean jerk differentiation for both cued and spoken expressions; mean activation, mean activation precision, mean activation differentiation for both cued and spoken expressions; plus AQ and TAS. We selected this machine learning approach in part due to the high degree of collinearity among several of our predictor variables; for example, mean cued activation and mean spoken activation were strongly correlated (*r* = 0.829, *p* < 0.001). Traditional regression methods assume low multicollinearity, and violation of this assumption can lead to inflated and unstable standard errors, unreliable *p* values, and an increased risk of both Type I and Type II errors (Hoffmann and Shafer 2015; Mason 1987; Mela and Kopalle 2002; Tu et al. 2004). Random forests, by contrast, are more robust to multicollinearity and can provide more stable estimates of variable importance under these conditions (Dormann et al. 2013; Tomaschek et al. 2018).

**FIGURE 12:**
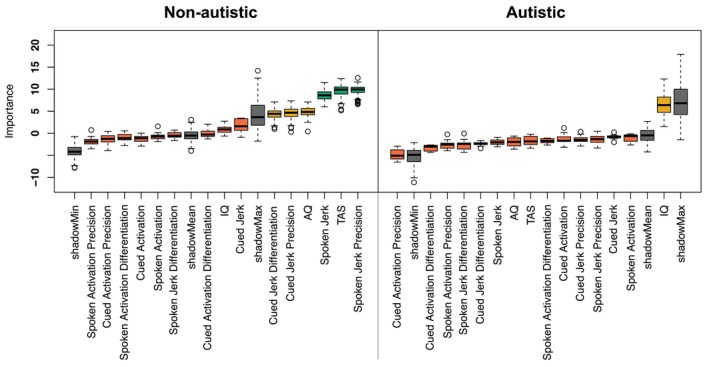
Random forest importance scores for non‐autistic (left) and autistic (right) emotion recognition. Boxplots show the importance of 15 features entered into the Boruta algorithm. Box edges represent the interquartile range (IQR), whiskers extend 1.5 * IQR, and circles mark outliers. Box color indicates decision: Green (confirmed), Yellow (tentative), Red (rejected). Gray represents meta‐attributes (shadowMin, shadowMax, shadowMean).

For the non‐autistic participants, of the 15 variables tested, three were classified as important, three as tentatively important, and nine were deemed unimportant for emotion recognition. Figure 12 (left) shows that spoken jerk precision [MIS = 9.75], TAS score [MIS = 9.54] and mean spoken jerk [MIS = 8.61] were classed as important for emotion recognition. AQ [MIS = 4.81], cued jerk precision [MIS = 4.61] and cued jerk differentiation [MIS = 4.37] were tentatively important for non‐autistic emotion recognition. All other variables were deemed unimportant. Notably, here we found that variables corresponding to the spoken condition were deemed important for emotion recognition, while those in the cued condition were deemed tentatively important. This finding is expected; participants may be more likely to draw on their own spoken productions since the stimuli in the emotion recognition task also comprise spoken expressions.

For the autistic participants, of the 15 variables tested, one was classified as tentatively important, and 14 were classified as unimportant for emotion recognition. As shown in Figure 12 (right), IQ was deemed tentatively important [MIS = 6.35] and all other variables were deemed unimportant for autistic emotion recognition.

Next, to verify the results from our random forests analyses, we conducted a linear regression in each group, predicting mean emotion recognition accuracy with the “important” and “tentatively important” variables. In these regressions, we added the predictor variables sequentially, starting with the variables with the highest mean importance scores, until there was no longer a significant improvement to the model. This follow‐up analysis was conducted to complement the strengths of the random forests approach. While random forests combined with the Boruta algorithm are well suited for robust feature selection—particularly in the context of multicollinearity and high‐dimensional data—they do not provide direct estimates of effect size, directionality, or statistical significance. Linear regression, by contrast, allows for clearer interpretation of the strength and direction of associations, variance explained, and statistical inference through *p* values. Thus, these regression models served to validate and clarify the relationships between the selected predictors and emotion recognition accuracy.

For non‐autistic individuals, entering mean spoken jerk precision as a predictor of emotion recognition significantly improved the model [*F* change = 16.59, *p* < 0.001, R^2^ change = 41.9%], accounting for 41.9% of the variance. Adding TAS score in the second step marginally improved the model [*F* change = 4.27, *p* = 0.051, R^2^ change = 9.9%], accounting for an additional 9.9% of the variance. There were no further improvements to the model when we added the remaining important and tentatively important variables. These results suggest that, for non‐autistic people, those with more precise spoken productions (with respect to jerk) tended to have greater emotion recognition accuracy.

To evaluate the strength of evidence for this model, we conducted Bayesian analyses separately for each group. Unlike traditional frequentist analyses, which only test whether an effect is statistically significant, Bayesian methods quantify the degree of evidence for both the presence and absence of an effect—providing a more nuanced assessment. This was particularly useful in our case, as it allowed us to determine whether the same predictors that were informative for non‐autistic individuals also explained variance in autistic individuals. The results showed very strong evidence for the model (where spoken jerk precision and alexithymia predict emotion recognition) in the non‐autistic group [BF_10_ = 87.55, *R* ^2^ = 51.4%], but moderate evidence for the *null* model in the autistic group [BF_10_ = 0.22, *R* ^2^ = 2.4%], supporting the idea that different factors may be linked to autistic and non‐autistic emotion recognition.

For autistic individuals, IQ was a significant positive predictor [*t* = 2.60, *b* = 0.48, *p* = 0.016], accounting for 22.6% of the variance in emotion recognition accuracy, and significantly improving the model [F change = 7.73, *p* = 0.016, *R* ^2^ change = 22.6%]. Bayesian analyses demonstrated that there was moderately strong evidence for this model relative to a null model [BF_10_ = 3.62]. In contrast, the same analysis demonstrated weak evidence for the null model for non‐autistic individuals [BF_10_ = 0.94, *R* ^2^ = 10.2%].

We also conducted additional analyses to ascertain (1) whether the autistic participants produced more or less precise and idiosyncratic facial expressions than their non‐autistic peers, (2) whether age, gender, or IQ were associated with mean levels of jerk or activation, (3) the (Bayesian) prevalence of our group effects, (4) individual differences that might be related to spoken jerk precision in the non‐autistic group, and (5) the contribution of autistic and alexithymic traits to the precision and differentiation of angry, happy and sad facial expressions. These analyses, which are outside of the scope of the main manuscript, are reported in Supporting Information F–K respectively.

## 4 Discussion

In this study, we first compared the facial expressions produced by autistic and non‐autistic individuals, after controlling for differences in facial morphology and alexithymia, and second, explored whether the jerkiness, activation, precision, and differentiation of participants' own emotional expressions contributed to their ability to recognize others'. Our results suggest that both autism and alexithymia contribute to levels of activation and jerk when producing emotional expressions, with these effects varying by emotion (i.e., across anger, happiness, and sadness), facial action unit (e.g., brow down blendshapes, mouth smile blendshapes, etc.), and posing condition (i.e., cued versus spoken). That is, compared to non‐autistic participants, the autistic participants did not show a consistent pattern of higher or lower activation or jerk across all facial features, emotions, and conditions. Instead, they displayed higher activation or jerk in some facial regions for certain emotions and posing conditions, lower activation or jerk in others, and in some cases, there were no differences between groups. This evidence points to some differences in both the configuration (i.e., relative activation) and kinematics of facial features between autistic and non‐autistic individuals when expressing emotion. Such mismatches could, at least partially, explain why autistic individuals find it difficult to recognize the emotions of non‐autistic people, and vice versa (Keating and Cook 2020; Brewer et al. 2016; Lampi et al. 2023; Love 1993); autistic and non‐autistic faces may be essentially “speaking a different language” when conveying emotion (Keating 2023). Therefore, what have previously been thought of as intrinsic emotion recognition “deficits” for autistic people may be more accurately described as difficulties resulting from cross‐neurotype interactions. Further research is needed to test the impact of expressive differences on emotion recognition for autistic and non‐autistic people.

For anger, across both conditions, the autistic participants displayed lower activation of the brow down blendshapes, and higher activation of specific mouth blendshapes (e.g., mouth frown, mouth upper up), than their non‐autistic peers (even after controlling for facial morphology and alexithymia). Autistic individuals also displayed significantly higher jerk for all mouth facial landmarks in the cued condition. Together, this evidence suggests that autistic people may rely more on the mouth, and less on the eyebrow region, to signal anger than their non‐autistic counterparts, both during cued and spoken emotional expressions. Interestingly, autistic individuals typically attend more to the mouth, and less to the eye region (than their non‐autistic peers), when *recognizing* emotional expressions (Klin et al. 2002; Riby et al. 2009; Calder et al. 2000). One possible explanation that develops from our current findings is that, since autistic individuals rely more on the mouth, and less on the eyebrows, (than non‐autistic individuals) to signal anger themselves, these participants may expect there to be more expressive information in the mouth region, and thus attend to this area more. Such attentional biases could then lead to downstream difficulties recognizing anger since the majority of expressive information is thought to be conveyed in the upper half of the face (Calder et al. 2000; Smith et al. 2005). Further research is necessary to test whether differences between groups in the *production* of emotional facial expressions contribute to differences in the *sampling* and *recognition* of them.

For happiness, there were large differences between groups in activation for both cued and spoken expressions, even after controlling for facial morphology and alexithymia. Specifically, the autistic participants displayed significantly lower activation of many blendshapes typically associated with happiness in both conditions—the left and right mouth smile, cheek squint, eye squint, and brow down blendshapes. By contrast, we found that the autistic participants exhibited higher activation for other cheek and mouth blendshapes (e.g., mouth funnel, mouth pucker, cheek puff, and mouth roll upper) in both conditions. Together, these results suggest there are group differences in mouth configuration when expressing happiness, with autistic individuals displaying a less exaggerated, and more puckered smile. Moreover, our results suggest that autistic participants rely less on the eyes, eyebrows, and cheeks than their non‐autistic peers when posing happiness. This may explain why autistic expressions have been rated as less natural in previous experiments (Faso et al. 2015): in the neurotypical literature, genuine (i.e., natural) happy expressions are said to be characterized by activation of *both* the zygomaticus major muscle—which pulls the lip corners upwards (i.e., mouth)—and the orbicularis oculi muscle—which lifts the cheeks, gathers the skin around the eye, and pulls the brow down—while non‐genuine happy expressions only involve the former (Ekman and Friesen 1982; Frank and Ekman 1993; Iwasaki and Noguchi 2016). Hence, autistic happy expressions may be perceived as less genuine (by non‐autistic observers), as they mostly involve activation of the zygomaticus major muscle (i.e., the mouth). Notably, although these expressions may be perceived as less genuine according to neurotypical criteria, this does not necessarily mean that autistic individuals produce less authentic or more forced expressions. Rather, it could be that genuine happy expressions for autistic individuals do not involve the orbicularis oculi to the same extent as for non‐autistic individuals. Further work is necessary to characterize genuine and posed autistic facial expressions, and to ascertain whether autistic expressions are rated as less natural or atypical in appearance (Faso et al. 2015; Grossman et al. 2013; Loveland et al. 1994; Macdonald et al. 1989) due to lower activation of the orbicularis oculi.

For sadness, there were fewer group differences in activation (relative to anger and happiness), and no group differences in jerk, after controlling for facial morphology and alexithymia. In the cued condition, the autistic participants exhibited significantly lower activation of the jaw forward blendshape at peak expression, and higher activation of the mouth upper up blendshape when transitioning into the expression. In the spoken condition, the autistic participants displayed significantly lower activation of the mouth frown, mouth roll, and eye squint blendshapes (at specific moments in time), but higher activation of the mouth upper up, brow outer up, and jaw left blendshapes. Thus, once again, our results point to different facial configurations for both cued and spoken sad expressions between groups. Most notably, the autistic participants tended to raise their upper lip more (cued and spoken condition), and pull the corners of their mouth down less (spoken condition), to display the downturned mouth that is characteristic of a sad expression (than their non‐autistic peers).

The results of the current study partially support our hypothesis concerning the jerkiness of facial movements. Based on prior research showing jerkier whole‐body, upper‐limb, and head movements [see Cook (2016)], we predicted that autistic participants here would display significantly more jerky facial expressions than their non‐autistic counterparts. However, whilst the autistic participants (relative to non‐autistic participants) exhibited higher jerk at all mouth landmarks for cued expressions of anger, thus supporting our hypothesis, we also found lower jerk at specific eyebrow landmarks for cued expressions of happiness, and no differences in jerk for sadness, contradicting our hypothesis. Moreover, there were no differences in jerk between groups in the spoken condition, in contrast to our hypothesis.

In this project, we found that alexithymia significantly contributed to the production of emotional facial expressions, both in terms of activation and jerk. For example, alexithymia, and not autism, was associated with less differentiated angry and happy facial expressions for both cued and spoken expressions. Specifically, in the spoken condition, we found that individuals high in alexithymia displayed elevated activation of the mouth smile blendshapes when posing anger, and the mouth frown blendshape when posing happiness. Concurrently, for both cued and spoken expressions, we found that there were smaller differences in activation between angry and happy facial expressions across blendshapes for those high, relative to low, in alexithymia. These results suggest that highly alexithymic individuals may produce more overlapping or ambiguous, angry and happy facial expressions. This challenges previous findings which have attributed less differentiated expressions in autistic individuals to autism, rather than alexithymia (Yirmiya et al. 1989; Rozga et al. 2013). Further research is needed to determine whether alexithymia leads to greater overlap between other emotional expressions (e.g., anger and disgust, surprise and fear, etc.), and to investigate whether observers have difficulty recognizing the less differentiated expressions of highly alexithymic individuals.

Another key aim of this study was to explore links between the production and perception of emotional expressions in autistic and non‐autistic individuals. Leveraging the body movement and emotional experience literatures we predicted that less precise and/or differentiated facial expressions would be associated with reduced emotion recognition accuracy. We found that precision was an important contributor for non‐autistic individuals, accounting for 41.9% of the variance in emotion recognition accuracy: those who produced highly variable spoken expressions (in terms of jerk) typically had poorer accuracy on an independent emotion recognition task. In a further exploratory analysis, we also identified a potential mechanistic pathway by which alexithymia contributes to emotion recognition difficulties (see Supporting Information I): alexithymia may lead to more variable productions of emotional expressions, which may in turn lead to greater emotion recognition difficulties (i.e., an indirect effect). Nevertheless, since mediation analyses cannot definitively determine causality (Bollen and Pearl 2013), future studies employing causal manipulation are necessary to confirm this.

While the precision of spoken productions predicted emotion recognition for non‐autistic individuals, no production‐related factors contributed to emotion recognition for autistic individuals. For this group, IQ was the only significant contributor, explaining 22.6% of the variance in accuracy. These results contribute to a growing literature suggesting that different psychological mechanisms are involved in autistic and non‐autistic emotion recognition (Keating et al. 2023, 2026; Rump et al. 2009; Rutherford and McIntosh 2007; Walsh et al. 2014). Within this literature, there is evidence that the precision of *visual emotion representations* (i.e., emotional expression in the “mind's eye”) contributes to emotion recognition accuracy for non‐autistic individuals, but not autistic individuals (Keating and Cook 2023; Keating et al. 2023). Taken together, these studies suggest that autistic individuals may not be using their visual representations and productions of facial expressions to help them recognize others' emotions (as much as their non‐autistic peers). This idea aligns with Bayesian theories of autism which propose that, compared to non‐autistic people, autistic individuals are less influenced by prior expectations (Lawson et al. 2014). In this framework, a visual representation of an emotion can be considered a *prior*—an internal prediction about what a given emotion should look like. While non‐autistic individuals may recognize emotions by comparing incoming sensory information (i.e., facial expressions) to this prior, autistic individuals may place less weight on such priors, potentially relying more on the incoming sensory information. This reduced influence of priors could help explain why the precision of one's own facial expressions is linked to emotion recognition for non‐autistic individuals but not for autistic individuals.

If autistic individuals rely less on stored visual representations and productions of facial expressions, how are they recognizing other people's emotions? One plausible explanation is that autistic individuals may have developed cognitively or verbally mediated compensatory strategies (Keating et al. 2023, 2026; Rump et al. 2009; Rutherford and McIntosh 2007; Walsh et al. 2014). For example, a “rule‐based” strategy where the incoming expression is matched to a list of features associated with different emotions (e.g., anger: “furrowed eyebrow”; happiness: “lips raised”; sadness: “downturned mouth”) (Rutherford and McIntosh 2007; Walsh et al. 2014). If autistic individuals are employing these cognitively or verbally mediated rule‐based strategies, then we might expect emotion recognition performance to be related more to verbal or cognitive ability in the autistic than non‐autistic group. Supporting this idea, here we found that IQ was a significant predictor of emotion recognition for the autistic [F (1,23) = 6.73, *p* = 0.013, *R* ^2^ = 22.6], but not non‐autistic participants [F (1,23) = 2.62, *p* = 0.120, *R* ^2^ = 10.2%]. Concurrently, if autistic individuals are employing more cognitive strategies, rather than automatically comparing to their visual representations or productions, this could also explain the longer emotion recognition response latencies found for autistic individuals (Georgopoulos et al. 2022; Hileman et al. 2011; Loth et al. 2018; McPartland et al. 2004; O'Connor et al. 2005; O'Connor et al. 2007; Webb et al. 2006). Further research is necessary to test whether autistic people adopt a rule‐based strategy to recognize others' emotions, and to identify what other factors contribute to autistic emotion recognition.

### 4.1 Strengths, Limitations and Future Directions

A key strength of this study is that we adopted a landmark detection approach—in which we extracted activation and jerk for numerous facial landmarks—rather than an emotion detection approach—wherein coarse estimations are computed regarding the extent to which anger, happiness, and/or sadness is displayed [e.g., FaceReader, Facet, FaceVideo; see (Dupré et al. 2020)]. This approach offers several advantages. First, it allowed us to move beyond capturing global differences in angry, happy, and sad facial expressions and instead comprehensively compare levels of activation and jerk across all facial features, for these emotions. This approach enabled us to disentangle mixed findings on emotional expressivity in autism, showing that expressions may appear more or less intense depending on the facial region being studied. Second, examining numerous facial features allowed us to identify what specifically is different about autistic and non‐autistic expressions, thus addressing a critical gap in the literature [see (Keating and Cook 2020)], and opening avenues to interventions aimed at enhancing cross‐neurotype emotions recognition. Third, our approach does not require software to assume, or make a prediction about, the emotion being displayed. This is pertinent given that previous work has critiqued the accuracy of the predictions made by automated emotion detection software (Burgess et al. 2023; Dupré et al. 2018; Küntzler et al. 2021).

While this study provides valuable insights into the differences in voluntarily produced facial expressions between autistic and non‐autistic individuals, further research is needed to characterize and compare spontaneous expressions. Here, we focused specifically on voluntary expressions, which are ubiquitous in everyday life, posed in order to deliberately communicate one's thoughts, intentions, and emotions to interaction partners (Parkinson 2005; Frith 2009; Jack and Schyns 2015). However, it is important to note that spontaneous expressions are also common in day‐to‐day life, may comprise more accurate indicators of an individual's emotions (Jia et al. 2021) and may be enervated via different pathways to (Rinn 1984; Morecraft et al. 2001) and look different from (Namba et al. 2017; Park et al. 2020) posed expressions. As such, the patterns we observed may not reflect how autistic and non‐autistic individuals express emotions in spontaneous, emotionally charged situations. It is also possible that autistic and non‐autistic people differ more in their ability to *deliberately pose* facial expressions than in their spontaneous productions, and thus we may overestimate expressive differences here. Supporting this possibility, there is evidence that autistic individuals produce less recognizable happy expressions (than their non‐autistic counterparts) only when posing, and not when expressions are naturalistically elicited (Faso et al. 2015). This suggests that there are differences in the appearance of posed and spontaneous happy expressions among autistic people. Thus, in sum, the findings documented here may not generalize to spontaneously produced emotional expressions. Future research should examine how autism and alexithymia contribute to the spatiotemporal and kinematic properties of spontaneous expressions.

Beyond this, further research is needed to compare the facial expressions produced by autistic and non‐autistic individuals for additional emotions, such as fear, disgust, and surprise. In the present study, we focused on anger, happiness, and sadness for both theoretical and practical reasons. Theoretically, these emotions were chosen based on previous work showing that autistic adults exhibited selective difficulties recognizing angry—but not happy or sad—facial expressions posed by non‐autistic people, relative to their non‐autistic peers (Keating et al. 2022). This raised the possibility that autistic and non‐autistic individuals might also differ more in their production of angry, compared to happy or sad, expressions—potentially contributing to the observed recognition differences. However, our findings did not support this hypothesis: the largest group differences emerged in the production of happy expressions. These three emotions were also selected because they span a broad range of affective space within the circumplex model of emotion (Russell 1980), representing both positive and negative valence as well as high (anger) and low (sadness) arousal. Moreover, they capture a wide portion of “expression‐space”—a conceptual extension of face‐space that accounts for variation in facial expressions of emotion (Calder et al. 2001). From a practical standpoint, including additional emotions (e.g., fear, disgust, and surprise) would have substantially lengthened the recording session, as multiple repetitions of each expression were required to ensure adequate statistical power. This was not feasible within the broader testing battery, and we were mindful that such tasks could be especially fatiguing for autistic participants, potentially introducing confounds related to exhaustion.

A further limitation concerns our sample size and composition. The relatively small group sizes mean we may not have fully captured the breadth of facial expressions produced by autistic and non‐autistic individuals. Autism is highly heterogeneous, with variability in genetics, neural systems, cognitive attributes, social communication, focused interests, and repetitive behaviors (Georgiades et al. 2013; Geurts et al. 2021; Cruz Puerto and Sandín Vázquez 2024; Qi et al. 2020), and is often accompanied by a range of co‐occurring conditions that further contribute to unique phenotypes (Hobson and Petty 2021). In the present study, we observed that this heterogeneity extends to facial expressions, with autistic participants producing more idiosyncratic expressions than their non‐autistic peers (see Supporting Information F). Given this heightened heterogeneity, the facial expressions recorded here may not fully represent the diversity of emotional displays within the autistic population. Future research with larger, more demographically representative samples will be essential for mapping the full range of expressive styles and for understanding how such heterogeneity influences emotion recognition both within and across neurotypes.

## Author Contributions

C.T.K. and S.S‐C. designed the study. C.T.K. collected the data, processed and analyzed the data, and wrote an initial draft. H.O.D. assisted with data‐processing. C.T.K. and J.L.C. reviewed and edited the initial draft. Supervision was conducted by J.L.C. All authors read and approved the final manuscript.

## Funding

This project was supported by the Medical Research Council (MRC, United Kingdom) MR/R015813/1 and the European Union's Horizon 2020 Research and Innovation Programme under ERC‐2017‐STG Grant Agreement No 757583.

## Disclosure

No materials are reproduced from other sources.

## Ethics Statement

This study was approved by the Science, Technology, Engineering and Mathematics (STEM) ethics committee at the University of Birmingham (ERN_16‐0281AP9D) and was conducted in accordance with the principles of the revised Helsinki Declaration. All participants provided informed consent before taking part.

## Conflicts of Interest

The authors declare no conflicts of interest.

## Supporting information


**Table S1:** Participants' ethnicity information.
**Figure S1:** A diagram illustrating the 68 facial landmarks tracked in the current study. These facial landmarks are based on those captured by OpenFace.
**Figure S2:** Graphs showing the *t*‐values for the significant group (top) and alexithymia (bottom) effects on jerk across facial landmarks and time for angry cued expressions. Positive values (e.g., orange, red) signify higher jerk in the autistic participants or a positive predictive relationship between jerk and alexithymia. Negative values (e.g., blue and purple) signify lower jerk in the autistic participants or a negative predictive relationship between jerk and alexithymia.
**Figure S3:** Graphs showing the *t*‐values for the significant group (top) and alexithymia (bottom) effects on jerk across facial landmarks and time for happy cued expressions. Positive values (e.g., orange, red) signify higher jerk in the autistic participants or a positive predictive relationship between jerk and alexithymia. Negative values (e.g., blue and purple) signify lower jerk in the autistic participants or a negative predictive relationship between jerk and alexithymia.
**Figure S4:** Graphs showing the *t*‐values for the significant group (top) and alexithymia (bottom) effects on jerk across facial landmarks and time for sad cued expressions. Positive values (e.g., orange, red) signify higher jerk in the autistic participants or a positive predictive relationship between jerk and alexithymia. Negative values (e.g., blue and purple) signify lower jerk in the autistic participants or a negative predictive relationship between jerk and alexithymia.
**Figure S5:** Graphs showing the *t*‐values for the significant group (top) and alexithymia (bottom) effects on jerk across facial landmarks and time for angry spoken expressions. Positive values (e.g., orange, red) signify higher jerk in the autistic participants or a positive predictive relationship between jerk and alexithymia. Negative values (e.g., blue and purple) signify lower jerk in the autistic participants or a negative predictive relationship between jerk and alexithymia.
**Figure S6:** Graphs showing the *t*‐values for the significant group (top) and alexithymia (bottom) effects on jerk across facial landmarks and time for happy spoken expressions. Positive values (e.g., orange, red) signify higher jerk in the autistic participants or a positive predictive relationship between jerk and alexithymia. Negative values (e.g., blue and purple) signify lower jerk in the autistic participants or a negative predictive relationship between jerk and alexithymia.
**Figure S7:** Graphs showing the *t*‐values for the significant group (top) and alexithymia (bottom) effects on jerk across facial landmarks and time for sad spoken expressions. Positive values (e.g., orange, red) signify higher jerk in the autistic participants or a positive predictive relationship between jerk and alexithymia. Negative values (e.g., blue and purple) signify lower jerk in the autistic participants or a negative predictive relationship between jerk and alexithymia.
**Figure S8:** A graph showing the inter‐participant variability in activation across blendshapes when posing an angry expression, for the autistic (green) and non‐autistic participants (purple).
**Figure S9:** A graph showing the inter‐participant variability in activation across blendshapes when posing a happy expression, for the autistic (green) and non‐autistic participants (purple).
**Figure S10:** A graph showing the inter‐participant variability in activation across blendshapes when posing a sad expression, for the autistic (green) and non‐autistic participants (purple).
**Table S2:** Relationships between our facial movement metrics and age and IQ, respectively.
**Figure S11:** Mediation models showing the contribution of alexithymia to non‐autistic emotion recognition via spoken jerk precision. The asterisks (*) denote statistical significance based on 95% confidence intervals.

## Data Availability

The data that support the findings of this study are openly available in Open Science Framework at https://osf.io/8a5yw/.
