# Supplementary material for: Mismatching Expressions: Spatiotemporal and Kinematic Differences in Autistic and Non‐Autistic Facial Expressions
Source: Autism Res. 2026 Jan 18;19(2):e70157. doi: 10.1002/aur.70157 (PMC12948747; doi:10.1002/aur.70157)
Supplement: Supplementary file 1 — Table S1: Participants' ethnicity information. Figure S1: A diagram illustrating the 68 facial landmarks tracked in the current study. These facial landmarks are based on those captured by OpenFace. Figure S2: Graphs showing the t‐values for the significant group (top) and alexithymia (bottom) effects on jerk across facial landmarks and time for angry cued expressions. Positive values (e.g., orange, red) signify higher jerk in the autistic participants or a positive predictive relationship between jerk and alexithymia. Negative values (e.g., blue and purple) signify lower jerk in the autistic participants or a negative predictive relationship between jerk and alexithymia. Figure S3: Graphs showing the t‐values for the significant group (top) and alexithymia (bottom) effects on jerk across facial landmarks and time for happy cued expressions. Positive values (e.g., orange, red) signify higher jerk in the autistic participants or a positive predictive relationship between jerk and alexithymia. Negative values (e.g., blue and purple) signify lower jerk in the autistic participants or a negative predictive relationship between jerk and alexithymia. Figure S4: Graphs showing the t‐values for the significant group (top) and alexithymia (bottom) effects on jerk across facial landmarks and time for sad cued expressions. Positive values (e.g., orange, red) signify higher jerk in the autistic participants or a positive predictive relationship between jerk and alexithymia. Negative values (e.g., blue and purple) signify lower jerk in the autistic participants or a negative predictive relationship between jerk and alexithymia. Figure S5: Graphs showing the t‐values for the significant group (top) and alexithymia (bottom) effects on jerk across facial landmarks and time for angry spoken expressions. Positive values (e.g., orange, red) signify higher jerk in the autistic participants or a positive predictive relationship between jerk and alexithymia. Negative values (e.g., blue a [file AUR-19-0-s001.docx]

| **Ethnicity** | **N** |
| --- | --- |
| Asian British | 2 |
| Asian Indian | 2 |
| Asian Pakistani | 1 |
| Black African | 1 |
| Black British | 1 |
| Black Caribbean | 2 |
| White British/English/Welsh/Scottish/Northern Irish | 40 |
| White European | 1 |
| White Polish | 1 |

**Supporting Information A – Participants’ ethnicity information.**

***Table S1.*** Participants’ ethnicity information.

**Supporting Information B – Analyses** **on raw emotion intensity ratings**

In the main manuscript, we found a significant relationship between spoken jerk precision and emotion recognition accuracy among the non-autistic participants. To gain further insight into this relationship, we conducted post-hoc linear models predicting mean spoken jerk precision from the emotion intensity ratings, for each of the 108 stimulus videos. For each of these models, we extracted the standardized beta coefficients reflecting the contribution of the correct and incorrect emotion ratings, respectively. Finally, we ran frequentist and Bayesian one-sample t-tests to examine whether these beta coefficients for the correct and incorrect emotion ratings were different from zero. Here, a deviation from zero in these beta values indicates an association between spoken jerk precision and the magnitude of emotion intensity ratings (for the non-autistic participants).

In these analyses, we found very strong evidence that the beta-coefficients reflecting the contribution of the correct emotion ratings were different from zero [t(107) = 4.21, p < .0001, BF_10_ = 326.16]. In contrast, there was moderate evidence that the beta-coefficients for the incorrect emotion ratings were not different from zero [t(215) = 1.38, p = .170, BF_10_ = 0.19]. These results suggest a significant relationship between mean spoken jerk precision and the magnitude of correct, but not incorrect emotion ratings for non-autistic people. That is, non-autistic individuals with more precise spoken expressions (in terms of jerk) tended to attribute higher ratings to the correct emotion (than those with less precise spoken expressions). This analysis offers further insight into the role of the precision (i.e., consistency) of one’s own productions in the appraisal and recognition of emotion.

**Supporting Information C – Order of facial blendshapes in our analyses.**

To see an illustration of these blendshapes, go to <https://arkit-face-blendshapes.com>

| **Order** | **Blendshape** |
| --- | --- |
| 1 | Basis |
| 2 | eyeBlinkLeft |
| 3 | eyeBlinkRight |
| 4 | eyeSquintLeft |
| 5 | eyeSquintRight |
| 6 | eyeWideLeft |
| 7 | eyeWideRight |
| 8 | jawForward |
| 9 | jawLeft |
| 10 | jawRight |
| 11 | jawOpen |
| 12 | mouthClose |
| 13 | mouthFunnel |
| 14 | mouthPucker |
| 15 | mouthLeft |
| 16 | mouthRight |
| 17 | mouthSmileLeft |
| 18 | mouthSmileRight |
| 19 | mouthFrownLeft |
| 20 | mouthFrownRight |
| 21 | mouthDimpleLeft |
| 22 | mouthDimpleRight |
| 23 | mouthStretchLeft |
| 24 | mouthStretchRight |
| 25 | mouthRollLower |
| 26 | mouthRollUpper |
| 27 | mouthShrugLower |
| 28 | mouthShrugUpper |
| 29 | mouthPressLeft |
| 30 | mouthPressRight |
| 31 | mouthLowerDownLeft |
| 32 | mouthLowerDownRight |
| 33 | mouthUpperUpLeft |
| 34 | mouthUpperUpRight |
| 35 | browDownLeft |
| 36 | browDownRight |
| 37 | browInnerUp |
| 38 | browOuterUpLeft |
| 39 | browOuterUpRight |
| 40 | cheekPuff |
| 41 | cheekSquintLeft |
| 42 | cheekSquintRight |
| 43 | noseSneerLeft |
| 44 | noseSneerRight |

**Supporting Information D – Full analytic procedures and justifications**

***Activation at the peak of cued expressions***

First, we aimed to determine whether there were group differences in activation during peak expression for anger, happiness, and sadness at specific blendshapes. Therefore, we extracted activation data at the midpoint of the expression (timepoint 270), for each blendshape, participant, and repetition, for each of the emotions respectively. This selection was based on both visual inspection and statistical analyses confirming that no other timepoints showed significantly higher activation. Following this, for each of the 44 blendshapes, we conducted a linear mixed effects model (LMMs) of activation as a function of group and TAS score, with subject and repetitions as random intercepts (816 datapoints for each model), for each of the emotions. This analytic approach was selected to appropriately account for the hierarchical structure of the data – namely, repeated blendshape activations nested within participants across multiple trials. LMMs enabled us to model both fixed effects (group and the covariate, alexithymia) and the random variability associated with individuals and repetitions. This is particularly important in repeated-measures designs, where traditional linear models may underestimate variance and inflate Type I error rates by ignoring within-subject variability (Baayen et al., 2008; Gueorguieva & Krystal, 2004). By using LMMs, we were able to obtain more accurate and generalisable estimates of group differences in facial expression activation (and later, jerk). In these linear mixed models, if we found a significant main effect of group, this would suggest that there are significant differences in activation between autistic and non-autistic individuals at the specific blendshape, even after controlling for alexithymia.

**Permutation-based approach for determining statistical significance**

To account for multiple comparisons, we carried out permutation tests (with 100 permutations). Within each permutation, the activation or jerk data for participants were shuffled so that they were randomly allocated to either the autistic or non-autistic group (and as such the data were shuffled amongst individuals with differing alexithymia scores). Following this, we conducted linear mixed models predicting shuffled activation or jerk at each blendshape/landmark with group, TAS score, and with subject and repetition as random intercepts (as above). The F values for the group and alexithymia effects on activation or jerk were extracted in each permutation. Next, all the F values for the shuffled data were sorted in order of magnitude. Finally, the effects in our analysis with true participant data were deemed to be significant if they exceeded the F value at the 95^th^ percentile (and thus alpha < .05) from the analysis with the shuffled data. Permutation testing is a well-established method for addressing multiple comparisons in fields such as neuroimaging, where effects are tested across numerous spatial and/or temporal units (e.g., voxels and/or timepoints), and where traditional corrections like Bonferroni are often overly conservative and can substantially reduce sensitivity (Groppe et al., 2011; Nichols & Holmes, 2002). By adopting this permutation-based approach, we were able to control the family-wise error rate while maintaining greater statistical power than would be possible with more conservative parametric corrections.

**Supporting Information E – Jerk across the time-course of cued and spoken expressions.**

*Cued condition.*

Here, we aimed to determine whether there were any differences between groups in the jerkiness of facial movements for anger, happiness, and sadness at specific landmarks and timepoints in the cued condition, after controlling for alexithymia. To test this, for each of the 68 landmarks (see Figure S1 for the location of these facial landmarks), at each of the timepoints, we conducted a linear mixed effects model of jerk as a function of group and TAS score, with subject and repetition as random intercepts, for each of the emotions. In these models, if we found a significant main effect of group, this would suggest that there are significant differences in jerk between autistic and non-autistic individuals at the specific landmark, at the specific moment in time, after controlling for alexithymia. As in the main manuscript, to account for multiple comparisons, we carried out a permutation test. Within each permutation, the jerk data for participants were shuffled so that they were randomly allocated to either the autistic or non-autistic group (and as such the data were shuffled amongst individuals with differing alexithymia scores). Following this, we conducted linear mixed models predicting (shuffled) jerk at each blendshape with group, TAS score, and with subject and repetition as random intercepts (as above). The F values for the group and alexithymia effects on jerk were extracted in each permutation. Next, all the F values for the shuffled data were sorted in order of magnitude. Finally, the effects in our analysis with true participant data were deemed to be significant if they exceeded the F value at the 95^th^ percentile (and thus alpha < .05) from the analysis with the shuffled data.

***Figure S1.*** A diagram illustrating the 68 facial landmarks tracked in the current study. These facial landmarks are based on those captured by OpenFace.

Our analysis identified that both autism and alexithymia contributed to the jerkiness of cued emotional expressions. Notably, however, these contributions varied as a function of the displayed emotion, the facial landmark, and timepoint. For anger, the autistic participants displayed higher jerk at all mouth facial landmarks at numerous timepoints throughout the expression (relative to their non-autistic peers). This explains why we found that the autistic participants exhibited higher jerk at this region when averaging across timepoints (see main manuscript). Moreover, our analysis revealed that the autistic participants displayed higher jerk at jaw, eyebrow, and nose landmarks when moving into and out of the angry expression (than the non-autistic participants; see Figure S2). Alexithymia was a positive predictor of jerk at some timepoints, and a negative predictor at other timepoints, across all the face areas (see Figure S2).

***Figure S2.*** Graphs showing the t-values for the significant group (top) and alexithymia (bottom) effects on jerk across facial landmarks and time for angry cued expressions. Positive values (e.g., orange, red) signify higher jerk in the autistic participants or a positive predictive relationship between jerk and alexithymia. Negative values (e.g., blue and purple) signify lower jerk in the autistic participants or a negative predictive relationship between jerk and alexithymia.

For happiness, the autistic participants displayed lower jerk at eye, eyebrow, nose, and jaw facial landmarks when moving into the out of the expression (relative to their non-autistic counterparts). The largest of these effects was for eyebrow landmarks; this explains why we found that the autistic participants exhibited lower jerk at this region when averaging across timepoints (see main manuscript). For mouth landmarks, the autistic participants exhibited higher jerk at some timepoints, and lower jerk at others (see Figure S3). As was the case for anger, alexithymia was a positive predictor of jerk at some timepoints, and a negative predictor at other timepoints, across all the face areas.

***Figure S3.*** Graphs showing the t-values for the significant group (top) and alexithymia (bottom) effects on jerk across facial landmarks and time for happy cued expressions. Positive values (e.g., orange, red) signify higher jerk in the autistic participants or a positive predictive relationship between jerk and alexithymia. Negative values (e.g., blue and purple) signify lower jerk in the autistic participants or a negative predictive relationship between jerk and alexithymia.

For sadness, the autistic participants displayed lower jerk at eyebrow, nose, and jaw landmarks at numerous timepoints when moving into, holding, and moving out of the expression (see Figure S4). At eye landmarks, the autistic participants displayed higher jerk at the start of the recording, and lower jerk when holding and moving out of the expression. Finally, the autistic participants displayed elevated jerk at mouth landmarks early in the expression. As was the case for anger and happiness, alexithymia was a positive predictor of jerk at some timepoints, and a negative predictor at other timepoints, across all the face areas.

***Figure S4.*** Graphs showing the t-values for the significant group (top) and alexithymia (bottom) effects on jerk across facial landmarks and time for sad cued expressions. Positive values (e.g., orange, red) signify higher jerk in the autistic participants or a positive predictive relationship between jerk and alexithymia. Negative values (e.g., blue and purple) signify lower jerk in the autistic participants or a negative predictive relationship between jerk and alexithymia.

*Spoken condition.*

Next, we aimed to determine whether there were any differences between groups in the jerkiness of facial movements for anger, happiness, and sadness at specific landmarks and timepoints in the spoken condition, after controlling for alexithymia. To test this, for each of the 68 landmarks, at each of the timepoints, we conducted a linear mixed effects model of jerk as a function of group and TAS score, with subject and repetition as random intercepts, for each of the emotions. As above, if we found a significant main effect of group, this would suggest that there are significant differences in jerk between autistic and non-autistic individuals at the specific landmark, at the specific moment in time, after controlling for alexithymia. To account for multiple comparisons, we conducted a permutation test to determine which effects were statistically significant (as described above).

Our analysis identified that both autism and alexithymia contributed to the jerkiness of spoken emotional expressions. Once again, these contributions differed across emotions, facial landmarks, and timepoints. When posing anger while speaking, the autistic participants displayed lower jerk at specific nose landmarks at numerous timepoints throughout the expression (relative to their non-autistic peers). Similarly, the autistic participants exhibited lower jerk at eye and jaw landmarks at select timepoints in the latter half of the expression (see Figure S5). As was the case for the cued expressions, alexithymia was a significant positive predictor of jerk at some timepoints, and a negative predictor at others, across all studied face areas (see Figure S5).

**

***Figure S5.*** Graphs showing the t-values for the significant group (top) and alexithymia (bottom) effects on jerk across facial landmarks and time for angry spoken expressions. Positive values (e.g., orange, red) signify higher jerk in the autistic participants or a positive predictive relationship between jerk and alexithymia. Negative values (e.g., blue and purple) signify lower jerk in the autistic participants or a negative predictive relationship between jerk and alexithymia.

When posing happiness while speaking, the autistic participants displayed lower jerk at early timepoints, and higher jerk at later timepoints, than their non-autistic peers. Specifically, the autistic participants exhibited lower jerk, particularly at eyebrow, nose, and jaw landmarks at early timepoints, and higher jerk at mouth and nose landmarks at later timepoints (see Figure S6). As previously, alexithymia was a significant positive predictor of jerk at some timepoints, and a negative predictor at others, across all studied face areas (see Figure S6). Most notably, alexithymia was a negative predictor of jerk at mouth landmarks at many timepoints early in the expression. This may explain why we identified alexithymia as a significant negative predictor of jerk for specific mouth landmarks when averaging across timepoints (see main manuscript).

***Figure S6.*** Graphs showing the t-values for the significant group (top) and alexithymia (bottom) effects on jerk across facial landmarks and time for happy spoken expressions. Positive values (e.g., orange, red) signify higher jerk in the autistic participants or a positive predictive relationship between jerk and alexithymia. Negative values (e.g., blue and purple) signify lower jerk in the autistic participants or a negative predictive relationship between jerk and alexithymia.

When posing sadness while speaking, the autistic participants displayed lower jerk at eye, mouth, and jaw landmarks at specific early (timepoints 120-170) and late (timepoints 210-300) timepoints (relative to their non-autistic counterparts). In contrast, the autistic participants exhibited elevated jerk at very early timepoints at both nose and mouth landmarks (approximately 40-110; see Figure S7). As previously, alexithymia was a significant positive predictor of jerk at some timepoints, and a negative predictor at others, across all studied face areas (see Figure S7). Of particular note, alexithymia was a strong negative predictor of jerk at mouth landmarks early in the expression. This may explain why we identified alexithymia as a significant negative predictor of jerk for specific mouth landmarks when averaging across timepoints (for sad spoken expressions).

***Figure S7.*** Graphs showing the t-values for the significant group (top) and alexithymia (bottom) effects on jerk across facial landmarks and time for sad spoken expressions. Positive values (e.g., orange, red) signify higher jerk in the autistic participants or a positive predictive relationship between jerk and alexithymia. Negative values (e.g., blue and purple) signify lower jerk in the autistic participants or a negative predictive relationship between jerk and alexithymia.

*Cued and spoken expressions: Summary*

In sum, there were significant differences between autistic and non-autistic individuals in the jerkiness of emotional facial movements at specific timepoints in both conditions. For cued angry expressions, the autistic participants displayed elevated jerk at all mouth facial landmarks throughout the expression; for cued happy expressions, the autistic participants displayed lower jerk at eye, eyebrow, nose, and jaw facial landmarks when moving into the out of the expression; for cued sad expressions, the autistic participants displayed lower jerk at eyebrow, nose, and jaw landmarks at numerous timepoints when moving into, holding, and moving out of the expression. For spoken expressions, while the autistic participants displayed higher jerk in some instances (e.g., nose for spoken sad expressions), generally, they tended to display lower jerk than their non-autistic counterparts at specific landmarks, such as the eyes (anger, sadness), eyebrows (happiness), nose (anger), and jaw (anger, happiness, sadness). Across emotions, alexithymia was a positive predictor of jerk at some timepoints and a negative predictor at others.

**Supporting Information F – Analyses to investigate whether (1) the autistic participants produced less precise expressions (i.e., more variable expressions across repetitions), and (2) the autistic participants produced more idiosyncratic expressions (i.e., more variable expressions across participants within their group).**

There are some suggestions that autistic individuals may produce more variable emotional facial expressions (across repetitions) than their non-autistic counterparts. Therefore, we constructed linear effects models to compare the autistic and non-autistic participants on cued activation precision, spoken activation precision, cued jerk precision, and spoken jerk precision, across emotions, after controlling for alexithymia (e.g., cued activation precision ~ emotion*group + alexithymia*group). In these analyses, there were no significant group effects or interactions [all p > .05], suggesting that the autistic and non-autistic participants produced comparably variable expressions (in terms of activation and jerk) across repetitions, for all three emotions, and in both conditions.

There are also suggestions that autistic individuals may produce more idiosyncratic emotional facial expressions than their non-autistic peers (see Brewer et al., 2016). To test this idea, we computed indices reflecting variability in activation across participants within the autistic and non-autistic groups, respectively, for each of the landmarks, at peak emotional expression (timepoint 270 in our cued condition). Subsequently, we constructed a linear mixed effects model of inter-participant variability as a function of emotion, group, and the emotion x group interaction, with facial blendshape as a random intercept. This analysis revealed a significant main effect of group [F(1,258) = 10.65, p = .001]: the autistic participants [mean(SEM) = 0.102(0.007)] produced more idiosyncratic expressions than their non-autistic peers [mean(SEM) = 0.081(0.006)]. There was no main effect of emotion [p = .215] nor an emotion x group interaction [p = .184]. In sum, these results suggest that the autistic participants produced more idiosyncratic expressions of anger, happiness, *and* sadness (i.e., expressions that are more variable and unique across participants within their group) than their non-autistic counterparts.

Next, we aimed to interrogate whether there are particular regions of the face where the autistic participants show idiosyncratic levels of activation. To test this, we first extracted the activation data at peak expression (i.e., at timepoint 270) for each blendshape, participant, and repetition, for each of the emotions respectively. Second, for each group respectively, we took the standard deviation in activation across participants, for each blendshape and repetition. These computations resulted in a matrix for each group, comprising 44 rows (corresponding to blendshapes) and 16 columns (corresponding to repetitions). The data within the cells of the matrix reflected inter-participant variability in activation at the specific blendshape, for that specific repetition. Following this, we conducted independent samples t-tests to test whether there were differences in inter-participant variability for each of the 44 blendshapes (across these repetitions). To account for multiple comparisons, we carried out a permutation test (with 100 permutations). Within each permutation, the inter-participant variability scores were shuffled such that they were randomly allocated to either the autistic or non-autistic group. Following this, we conducted independent samples t-tests to test whether there were differences in inter-participant variability scores between the shuffled autistic data and non-autistic data. We extracted the t values reflecting these differences in each permutation. Next, we took the absolute of all t values for the shuffled data, and sorted them in order of magnitude. Finally, the (absolute) t values from our analysis with true participant data were deemed to be significant if they exceeded the (absolute) t value at the 95^th^ percentile (and thus alpha < .05) from the analysis with the shuffled data (as in the main manuscript).

This analysis identified that there were significant group differences in inter-participant variability for the angry [68.18% blendshapes], happy [84.09% of blendshapes], and sad expressions [52.27% of blendshapes]. For anger, the autistic participants produced more idiosyncratic expressions than their non-autistic peers, with more variable levels of activation across participant for the left and right eye squint [left t = -11.06; right t = -11.06], eye wide [left t = -4.94; right t = -4.90], mouth [left t = -5.08; right t = -3.26], mouth smile [left t = -5.27; right t = -6.77], mouth frown [left t = -3.86; right t = -3.49], mouth stretch [left t = -12.55; right t = -12.57], mouth press [left t = -3.28; right t = -3.47], mouth lower down [left t = -6.43; right t = -6.11], mouth upper up [left t = -8.43; right t = - 8.74], brow down [left t = -12.21; right t = -12.26], brow outer up [left t = -5.95; right t = -6.00], and nose sneer [left t = -2.57; right t = -3.13] blendshapes, along with the jaw forward [t = -3.18], mouth close [t = -5.11], mouth funnel [t = -2.75], mouth shrug upper [t = -5.15], brow inner up [t = -4.52], and the cheek puff [t = -2.61] blendshapes (see Figure S8).


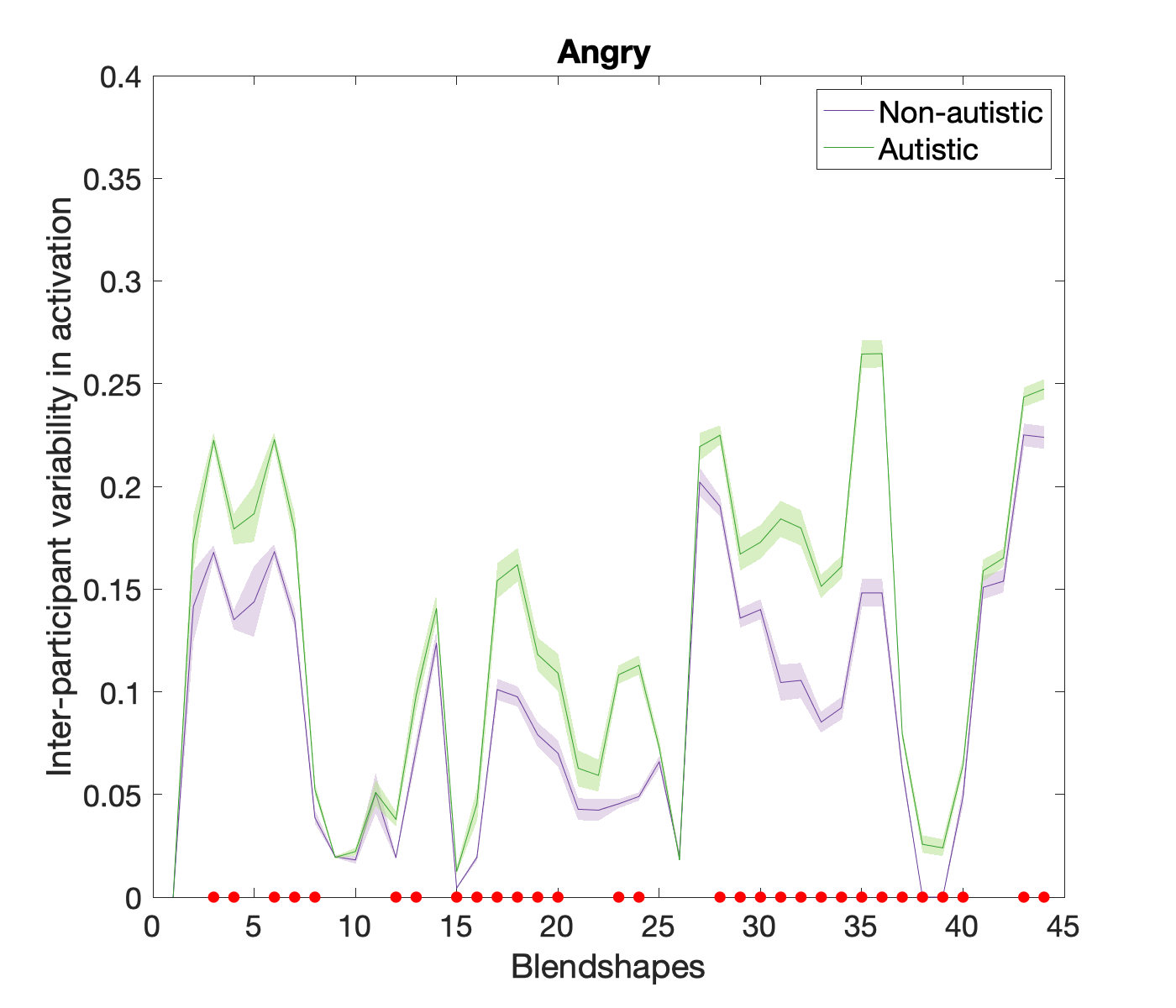


***Figure S8.*** A graph showing the inter-participant variability in activation across blendshapes when posing an angry expression, for the autistic (green) and non-autistic participants (purple).

The line on the graph shows the mean levels of inter-participant variability (across repetitions), and the shaded region corresponds to the standard error of the mean. Significant group effects are indicated by red dots on the graph.

For happiness, the autistic participants generally produced more idiosyncratic expressions than the non-autistic participants, with more variable levels of activation across participants for the left and right eye squint [left t = -2.62; right = -2.63], eye wide [left = -4.59; right = -4.49], mouth smile [left t = -13.57; right = -12.30], mouth press [left t = -8.61; right t = -8.89], mouth lower down [left t = -4.56; right = -3.97], brow down [left t = -3.49; right t = -3.47], brow outer up [left = -8.77; right = -8.77], cheek squint [left t = -7.88; right t = -7.36], and nose sneer blendshapes [left = -5.67; right = -5.47], along with the lower and upper mouth roll [lower t = -4.59; upper t = -5.37], the upper mouth shrug [t = -3.82], the mouth close [t = -10.12], mouth funnel [t = -9.13], mouth pucker [t = -7.90], right jaw [t = -2.58], right mouth [t = -3.59], and brow inner up [t = -9.78] blendshapes. By contrast, the autistic participants showed less variable levels of activation across participants (relative to their non-autistic peers) for the left and right mouth dimple [left t = 6.85; right t = 6.55], mouth stretch [left t = 4.66; right = 4.08], and mouth upper up [left t = 6.49; right t = 6.58] blendshapes, along with the jaw forward [t = 3.98], left jaw [t = 3.10], and jaw open [t = 2.22] blendshapes (see Figure S9).


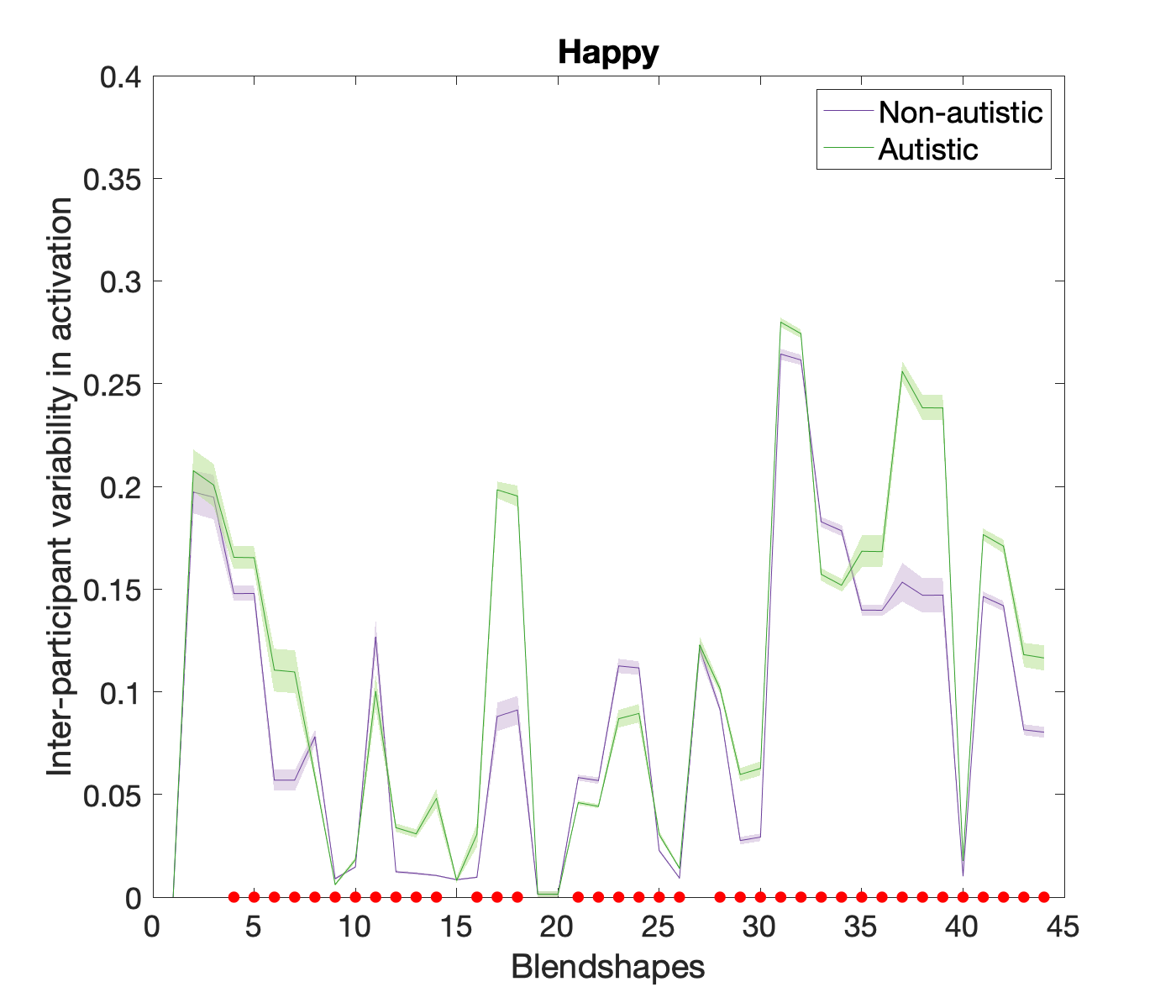


***Figure S9.*** A graph showing the inter-participant variability in activation across blendshapes when posing a happy expression, for the autistic (green) and non-autistic participants (purple).

The line on the graph shows the mean levels of inter-participant variability (across repetitions), and the shaded region corresponds to the standard error of the mean. Significant group effects are indicated by red dots on the graph.

For sadness, the autistic participants generally produced more idiosyncratic expressions than their non-autistic counterparts, with more variable levels of activation across participants for the left and right eye wide [left t = -6.93; right t = -6.91], mouth press [left t = -3.78; right t = -4.12], mouth lower down [left t = -3.21; right t = -3.29], and brow down [left t = -7.82; right = -7.78] blendshapes, along with the lower and upper mouth shrug [lower t = -3.66; upper t = 3.52], the mouth pucker [t = -12.31], jaw open [t = -4.16], left jaw [t = -6.01], and cheek puff [t = -5.31] blendshapes. Conversely, the autistic participants showed less variable levels of activation across participants (relative to their non-autistic peers) for the left and right mouth frown [left t = 13.13; right t = 13.43], mouth dimple [left t = 2.35; right = 2.63], and brow outer up [left t = 2.90; right t = 2.90] blendshapes, along with the jaw forward [t = 6.44], right jaw [t = 2.93], and brow inner up [t = 2.95] blendshapes (see Figure S10).


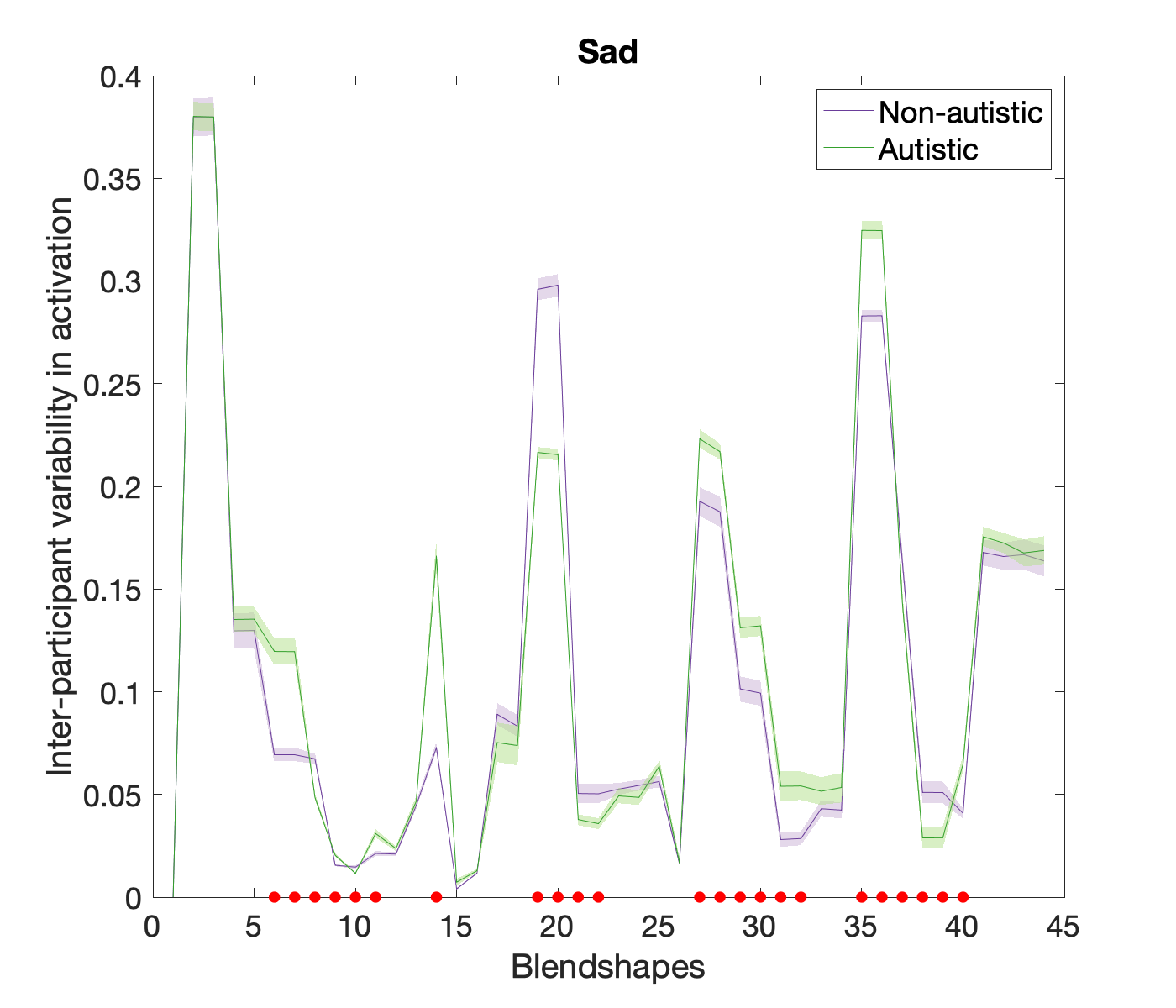


***Figure S10***. A graph showing the inter-participant variability in activation across blendshapes when posing a sad expression, for the autistic (green) and non-autistic participants (purple).

The line on the graph shows the mean levels of inter-participant variability (across repetitions), and the shaded region corresponds to the standard error of the mean. Significant group effects are indicated by red dots on the graph.

In sum, compared to their non-autistic peers, overall, the autistic participants produced more idiosyncratic angry, happy, and sad expressions, with more variable levels of activation (across participants) for a moderate-large proportion of the blendshapes.

**Supporting Information G – Examining the link between facial movements and age, gender and IQ**

Since there are suggestions that men and women differ in emotional expressiveness (e.g., Kring & Gordon, 1998), we conducted two post-hoc ANOVAs to investigate potential sex differences in levels of facial expression activation. The first analysed mean activation scores using a mixed 2 x 2 x 3 design with sex as a between-subjects factor and condition (cued, spoken) and emotion (angry, happy, sad) as within-subjects factors. The second ANOVA extended this to include group (autistic, non-autistic) as an additional between-subjects factor. Across both analyses, no significant main effects or interactions with sex were found [all p > .05], indicating no differences in expressivity between males and females for anger, happiness and sadness, across both conditions, and in both groups.

Next, we conducted two post-hoc ANOVAs to investigate potential sex differences in levels of facial movement jerk. The first analysed mean jerk scores using a mixed 2 x 2 x 3 design with sex as a between-subjects factor and condition (cued, spoken) and emotion (angry, happy, sad) as within-subjects factors. The second ANOVA extended this to include group (autistic, non-autistic) as an additional between-subjects factor. Across both analyses, no significant main effects or interactions with sex were found [all p > .05], indicating no differences in facial movement jerk between males and females for anger, happiness and sadness, across both conditions, and in both groups.

Finally, we conducted spearman’s correlations to examine whether our facial movement metrics were associated with age or IQ. Specifically, we tested whether participants' mean levels of facial activation or jerk were related to these demographic variables. The analysis revealed a significant positive correlation between age and mean facial activation in the cued condition [r = .350, p = .012]. However, this effect did not remain significant after applying a Bonferroni correction for multiple comparisons [p_bonf_ = .096]. There were no other significant relationships.

***Table S2.*** Relationships between our facial movement metrics and age and IQ, respectively.

|  | Mean Cued Jerk | Mean Spoken Jerk | Mean Cued Activation | Mean Spoken Activation |
| --- | --- | --- | --- | --- |
| Age | r = .172, p = .228 | r = .028, p - .844 | r = .350, p = .012, | r = .154, p = .282 |
| IQ | r = -.076, p = .598 | r = -.091, p = .527 | r = -.138, p = .333 | r = -.075, p = .601 |

**Supporting Information H – Bayesian inference of the prevalence of our group effects**

The conventional approach for evaluating data from multiple experimental groups involves null hypothesis statistical testing (NHST) on population means – that is, the average value in sampled populations (Ince et al., 2021; 2022). For example, in the main manuscript, we have conducted NHST to ask whether the observed mean from the autistic group significantly differs from that of the non-autistic group. While this approach provides valuable insights, focusing solely on population means can obscure the substantial within-group variability often seen in the field of psychology (Ince et al., 2021; 2022) and more specifically here, in the autistic population (see Masi et al., 2017).

To overcome this shortcoming, Ince et al., (2021, 2022) argue that researchers should focus on effects within individual participants, in a statistical framework known as Bayesian prevalence. In this framework, it is possible to quantify how prevalent an observed effect is within a given population, and the uncertainty around this estimate (Ince et al., 2021; 2022). For example, one can quantify the proportion of individuals within an autistic sample that are likely to display a given repetitive behaviour, sensory sensitivity, or motor atypicality. This approach is particularly valuable in autism research as (1) it enables researchers to quantify and gain insight into the heterogenous behavioural phenotypes of autistic people (see Masi et al., 2017 for a discussion on heterogeneity), (2) it allows researchers to identify subgroups which display certain characteristics – that may not be identified in whole population analyses (e.g., particular sensory sensitivities) – and then examine the factors underlying these characteristics within the subgroup (e.g., differences in interoception), and (3) it can help researchers to create more refined screening tools and diagnostic criteria which focus on the most prevalent characteristics. For example, in the domain of movement, taking a Bayesian prevalence approach may allow us to develop movement-based screening tools (e.g., see Crippa et al., 2015; Perochon et al., 2023; Keating & Cook, 2025) which focus on the most prevalent motor atypicalities. Moreover, determining the proportion of autistic people that show a given movement atypicality – such as finding that 70% of this group use their mouth rather than their eyebrows to signal anger – proves beneficial in aiding caregivers to interpret autistic facial expressions and respond appropriately. In sum, a Bayesian prevalence approach is fruitful here for quantifying the proportion of participants that showed particular movement atypicalities.

Therefore, here, we conducted analyses to demonstrate the Bayesian prevalence of our effects, following the procedures of Ince et al., (2021). This method estimates the proportion of the wider population likely to exhibit a given effect in the same experiment (the Bayesian maximum a posteriori (MAP) estimate), and identifies the range with 96% confidence that contains the true prevalence (96% Highest Posterior Density Interval (HPDI); Jaswal et al., 2024; Ince et al., 2021). Taking inspiration from the procedures of Ince et al., (2021), for each of the autistic participants (N = 25), we conducted a one-sample t-test to examine whether activation for the right mouth dimple blendshape across repetitions (N = 16), when posing happiness, was significantly lower than the non-autistic mean, at p < .05. We focused on this specific effect because it is comprised the average reported group difference in activation according to effect size [F = 6.82]. Via this analysis, we identified that 18 of the 25 autistic participants showed significantly lower activation at this blendshape, when posing happiness, relative to their non-autistic peers. The MAP estimate for this effect was 0.71, and the 96% HPDI was [0.50 0.86]). Thus, one could expect, with 96% confidence that between 50% and 86% of the autistic population would show significantly lower activation than their non-autistic counterparts, at this blendshape when posing happiness. In sum, given that the vast majority of our autistic sample (approximately 70%) showed this motor atypicality – which comprises the average sized effect from our main analyses – it is highly likely that the atypicalities we have reported would be present in at least a moderate proportion of the wider autistic population.

**Supporting Information I – The link between production and perception: Exploratory analyses**

In the main manuscript, our analyses implicated spoken jerk precision in emotion recognition within the *non-autistic* group. To gain insight into individual differences that might be related to spoken jerk precision in the non-autistic population, we conducted a further exploratory analysis. Specifically, we constructed a Bayesian linear regression model predicting spoken jerk precision with age, IQ, AQ, and TAS. The strongest model included only TAS as a predictor [BF_10_ = 5.44, R^2^ = 25.0%]: those higher in alexithymic traits typically displayed less precise (i.e., more variable) facial expressions in terms of jerk [t =-2.83, *b* = -0.50, p = .009]. Building on this we conducted an exploratory mediation analysis to test the hypothesis that alexithymia might exert an indirect effect on emotion recognition by influencing spoken jerk precision. This analysis revealed that there were both significant direct [z = -2.20, p = .028, 95% CI = (-0.051, -0.003)] and indirect [z = -2.10, p = .036, 95% CI = (-0.034, -0.001)] effects of alexithymia on emotion recognition accuracy (see Fig. S11). Hence our findings suggest a potential causal direction: for non-autistic individuals, being high in alexithymic traits may lead to more variable productions of emotional facial expressions, which in turn may result in poorer emotion recognition accuracy (in addition to a more direct effect).

**Figure S11.** Mediation models showing the contribution of alexithymia to non-autistic emotion recognition via spoken jerk precision. The asterisks (*) denote statistical significance based on 95% confidence intervals.

**Supporting Information J – The contribution of AQ and TAS to the differentiation of emotional expressions**

In the main manuscript, we reported that individuals with higher alexithymic traits tended to produce less distinct angry and happy expressions in both the cued and spoken conditions (in terms of activation). Next, we conducted exploratory analyses to examine whether alexithymic or autistic traits predicted the differentiation between angry and sad expressions, and between happy and sad expressions (in terms of activation), across both conditions. To address this question, we conducted exploratory random forest analyses (Breiman, 2001) using the Boruta Wrapper algorithm (Kursa & Rudnicki, 2010), focusing on the activation data.

***Differentiation of happy and sad expressions***

In these analyses, alexithymia was deemed important, and autism was deemed unimportant, for the differentiation of happy and sad expressions in the spoken condition [alexithymia MIS = 10.37; autism MIS = 2.12]. Those higher in alexithymia tended to produce less differentiated happy and sad expressions in the spoken condition. For the cued condition, both autism [MIS = -6.39] and alexithymia [MIS = 3.99] were deemed unimportant for the differentiation of happy and sad expressions.

***Differentiation of angry and sad expressions***

In these analyses, autism [MIS = 5.70] was deemed tentatively important, and alexithymia was unimportant [MIS = -1.12] for the differentiation of angry and sad expressions in the cued condition. Follow-up linear regressions confirmed that AQ was not a significant predictor of the differentiation of angry and sad expressions [p > .05]. For the spoken condition, both autism [MIS = 1.16] and alexithymia [MIS = -1.99] were deemed unimportant for the differentiation of angry and sad expressions.

**Supporting Information K – The contribution of AQ and TAS to the precision of emotional expressions**

Next, we conducted exploratory analyses to examine whether autistic or alexithymic traits contributed to the precision of angry, happy and sad facial expressions (in terms of activation) across both conditions. To address this question, we conducted exploratory random forest analyses (Breiman, 2001) using the Boruta Wrapper algorithm (Kursa & Rudnicki, 2010), focusing on the activation data. In these analyses, autism and alexithymia were both deemed unimportant for the precision of cued angry [autism MIS = 3.66; alexithymia MIS = 0.12], cued happy [autism MIS = 2.77; alexithymia MIS = 3.42], cued sad [autism MIS = -2.03; alexithymia MIS = -0.74], spoken happy [autism MIS = -1.83; alexithymia MIS = -0.98], and spoken sad [autism MIS = -2.60; alexithymia MIS = 1.05] expressions. In contrast, alexithymia was deemed tentatively important [MIS = 6.63] and autism was deemed unimportant [MIS = 1.88] for the precision of spoken angry expressions. Notably, however, a follow-up linear regression found that alexithymia was not a significant predictor of the precision of spoken angry expressions [all p > .05].

References

Baayen, R. H., Davidson, D. J., & Bates, D. M. (2008). Mixed-effects modeling with crossed random effects for subjects and items. Journal of Memory and Language, 59(4), 390–412.

Breiman, L. (2001). Random forests. *Machine learning*, *45*(1), 5-32.

Brewer, R., Biotti, F., Catmur, C., Press, C., Happé, F., Cook, R., & Bird, G. (2016). Can neurotypical individuals read autistic facial expressions? Atypical production of emotional facial expressions in autism spectrum disorders. *Autism Research*, *9*(2), 262-271.  <https://doi.org/10.1002/aur.1508>

Crippa, A., Salvatore, C., Perego, P., Forti, S., Nobile, M., Molteni, M., & Castiglioni, I. (2015). Use of machine learning to identify children with autism and their motor abnormalities. *Journal of autism and developmental disorders*, *45*, 2146-2156. <https://doi.org/10.1007/s10803-015-2379-8>

Groppe, D. M., Urbach, T. P., & Kutas, M. (2011). Mass univariate analysis of event-related brain potentials/fields I: A critical tutorial review. Psychophysiology, 48(12), 1711–1725.

Gueorguieva, R., & Krystal, J. H. (2004). Move over ANOVA: Progress in analyzing repeated-measures data and its reflection in papers published in the Archives of General Psychiatry. Archives of General Psychiatry, 61(3), 310–317.

Keating, C. T., & Cook, J. (2025). Facial movements as biomarkers for autism: A Bayesian prevalence and machine-learning proof-of-concept study. *PsyArXiv.* <https://doi.org/10.31234/osf.io/h4yd7_v1>

Kring, A. M., & Gordon, A. H. (1998). Sex differences in emotion: expression, experience, and physiology. *Journal of personality and social psychology*, *74*(3), 686. [https://doi.org/10.1037/0022-3514.74.3.686](https://psycnet.apa.org/doi/10.1037/0022-3514.74.3.686)

Kursa, M. B., & Rudnicki, W. R. (2010). Feature selection with the Boruta package. *Journal of statistical software*, *36*, 1-13.

Ince, R. A., Kay, J. W., & Schyns, P. G. (2022). Within-participant statistics for cognitive science. *Trends in cognitive sciences*, *26*(8), 626-630. <https://doi.org/10.1016/j.tics.2022.05.008>

Ince, R. A., Paton, A. T., Kay, J. W., & Schyns, P. G. (2021). Bayesian inference of population prevalence. *Elife*, *10*, e62461. <https://doi.org/10.7554/eLife.62461>

Jaswal, V. K., Lampi, A. J., & Stockwell, K. M. (2024). Literacy in nonspeaking autistic people. *Autism*, 13623613241230709. [https://doi.org/10.1177/1362361324123070](https://doi.org/10.1177/13623613241230709)

Masi, A., DeMayo, M. M., Glozier, N., & Guastella, A. J. (2017). An overview of autism spectrum disorder, heterogeneity and treatment options. *Neuroscience bulletin*, *33*, 183-193. <https://doi.org/10.1007/s12264-017-0100-y>

Perochon, S., Di Martino, J. M., Carpenter, K. L., Compton, S., Davis, N., Eichner, B., ... & Dawson, G. (2023). Early detection of autism using digital behavioral phenotyping. *Nature Medicine*, *29*(10), 2489-2497. <https://doi.org/10.1038/s41591-023-02574-3>

Nichols, T. E., & Holmes, A. P. (2002). Nonparametric permutation tests for functional neuroimaging: A primer with examples. Human Brain Mapping, 15(1), 1–25.
